# Complex IIa formation and ABC transporters determine sensitivity of OSCC to Smac mimetics

**DOI:** 10.1038/s41419-024-07253-w

**Published:** 2024-11-22

**Authors:** Yuhan Wang, Zijian Liu, Qian Si, Wanqiu Lu, Yuxian Song, Wanyong Jin, Xihu Yang, Zihui Li, Xinyang Hu, Liang Ding, Yue Jing, Pei Weng, Qiuya Yu, Lorraine A. O’Reilly, John Silke, Xiaoxin Zhang, Qingang Hu, Yanhong Ni

**Affiliations:** 1 Central Laboratory of Stomatology, Nanjing Stomatological Hospital, Affiliated Hospital of Medical School, Research Institute of Stomatology, Nanjing University, Nanjing, China; 2 Department of Oral and Maxillofacial Surgery, Nanjing Stomatological Hospital, Affiliated Hospital of Medical School, Nanjing University, Nanjing, China; 3 School of Biopharmacy, China Pharmaceutical University, Nanjing, Jiangsu China; 4 Department of Oral and Maxillofacial Surgery, Affiliated Hospital of Jiangsu University, Zhenjiang, China; 5 The Walter and Eliza Hall Institute of Medical Research, Parkville, VIC 3052 Australia; 6 Department of Medical Biology, University of Melbourne, Parkville, VIC 3010 Australia

**Keywords:** Cancer, Diseases

## Abstract

Small molecule inhibitors of apoptosis proteins (IAPs) antagonists, known as Smac mimetics (SMs), activate non-canonical NF-κB and sensitize cancer cells to TNF-induced cell death. SMs are currently in phase III clinical trials for head and neck squamous cell carcinoma (HNSCC) after promising phase II trials. To explore the utility of SMs in oral squamous cell carcinoma (OSCC), we tested nine human OSCC cell lines and correlated SM sensitivity with both IAP mutation and expression levels. cIAP1 protein expression was shown to be higher in OSCC and a predictor of poor prognosis. However, our in vitro and in vivo testing demonstrated differential sensitivity to SMs, which did not correlate with cIAP1 and cIAP2 expression in these OSCC cell lines. Exogenous TNF failed to effectively increase the sensitivity of SM-resistant OSCC cells to SM-induced cell death. SM resistance was associated with a deficiency in Complex IIa formation, but activation of non-canonical NF-κB was not a determinant of SM efficacy. Finally, metabolic analysis revealed that the ABC transporter pathway was activated in SM-resistant OSSC cells, and SMs combined with ABC transporter inhibitors improved cell death sensitivity to overcome SM resistance. These studies highlight the therapeutic potential of SMs in OSCC and support patient stratification to improve efficacy with the addition of adjuvant therapy.

## Introduction

The most common sub-group of head and neck squamous cell carcinoma (HNSCC) and the most commonly diagnosed malignancy of the oral cavity is oral squamous cell carcinoma (OSCC) [1, 2]. OSCC is a global health problem, with an increasing incidence in recent years [3, 4] and a mortality rate that continues to rise, particularly in the older female population (over 65 years) [5]. The standard of care for locally advanced OSCC and recurrent/metastatic OSCC is surgery combined with chemo-radiotherapy [6], but the 5-year overall survival rate has not improved for decades [7]. In addition, monoclonal antibodies, such as cetuximab (epidermal growth factor receptor inhibitor) have improved advanced HNSCC prognosis when combined with radiotherapy [8], but this combinative therapy was less effective than traditional chemoradiation [9]. First-line pembrolizumab (anti-PD-1 immune checkpoint inhibitor) with/without chemotherapy prolongs survival in populations with recurrent/metastatic HNSCC [10]. However, the high rate of adverse events (53.3%) [11] and failure to significantly improve efficacy [12] were reported in OSCC clinical trials. Therefore, there is a pressing need to explore new therapeutics for OSCC.

SMAC mimetics (SMs) are small peptide-like molecules, that mimic the action of the SMAC protein, the endogenous antagonist of inhibitors of apoptosis proteins (IAPs) [13]. Since cIAPs play an important role in activating the TNF-associated survival pathway, their antagonists sensitize cells to TNF-induced cell death [14, 15]. Therefore, the anti-cancer activity of SMs is closely correlated to TNF signaling. Apart from TNF-mediated extrinsic apoptosis, SMs also potentially sensitize cells to the intrinsic apoptotic pathway. Upon receipt of an intrinsic apoptotic signal, Bax and Bak oligomerize in the outer mitochondrial wall to form a pore that permits SMAC to enter the cytosol and bind to and inhibit IAPs, particularly XIAP, the caspase-3 caspase-9 inhibitor [16, 17]. Nevertheless, HNSCC is associated with mutations in genes such as cIAP1 [18]/BIRC2 [19], caspase-8 [20], FADD [19], and TNF [21] that affect the extrinsic apoptosis pathway indicating that this is pathway is likely to be particularly relevant when exploring SM induced cell death in HNSCC.

Due to the good safety profile and promising anti-tumor effects, especially in certain murine transplantation models [22–24], multiple clinical trials of SMs are underway. To date, SMs including GDC-0152, GDC-0917, AT406, LCL161, BI 891065, birinapant, APG-1387, ASTX660, and HGS1029 have been evaluated in clinical trials for various solid and hematological cancers [13, 25]. However, as single agents, SMs rarely induce complete responses in patients and a number of clinical trials have been terminated due to lack efficacy [26, 27]. However, as adjuvants to improve sensitivity to radiotherapy, chemotherapy, and immunotherapy, SMs are proving to be more beneficial [13]. Therefore, defining the mechanisms of impaired anti-tumor activities in cancer cells, resulting in clinical trial failure is urgently required.

Notably, SM-induced cell death also appears to be dependent on the tumor molecular subtype. For example, our previous research had demonstrated that SMs were more effective in triple-negative breast cancers than in other subtypes [28]. Moreover, in colorectal cancers, SMs had shown to increase the sensitivity of Consensus Molecular Subtypes 4 (CMS4) cells to oxaliplatin/5-FU, whereas CMS2 cells were unresponsive [29, 30]. This indicated that the molecular classification of patients may enhance the efficacy of SMs in clinical trials.

Clinical trials with SMs, including AT406, birinapant, APG-1387, and ASTX660 have been conducted in HNSCC, where safety was proven in phase I trials [31]. Of these, AT406 has commenced recruiting for HNSCC phase III clinical trials (NCT04459715, NCT05386550), since the encouraging results of the Phase II trial [32, 33]. Unfortunately, the latter trial included very few OSSC patients. Therefore, it is imperative to combine the clinicopathological characteristics of OSCC patients with molecular factors that may confer therapy sensitivity or resistance to assess the future feasibility of SM treatment for OSCC.

In order to define markers of SM sensitivity, this study assessed the expression of SM targets, namely cIAP1 and cIAP2, in OSCC cell lines and patients. Furthermore, this study compared the expression levels of key proteins and metabolic profiles in SM-sensitive and SM-resistant cells. While several known SM sensitivity factors, such as non-canonical NF-κB, did not correlate with sensitivity, we found that elevated levels of ABC family members (ABCA3, ABCB1, and ABCB4) and failure of Complex IIa formation contributed to SM resistance in OSCC.

## Results

### cIAP1 is aberrantly expressed in OSCC and predicts poor clinical outcome

**Fig. 1:**
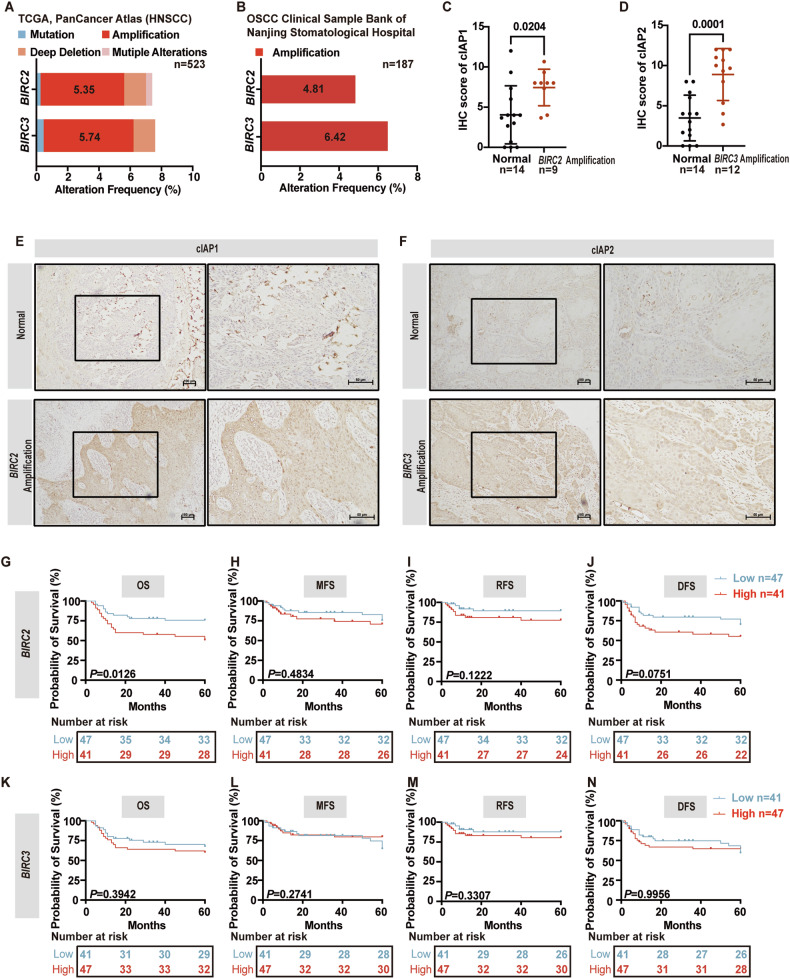
*BIRC2* and *BIRC3* mutations in OSCC and HNSCC patients. **A** Bar graph showing *BIRC2* and *BIRC3* DNA alterations in HNSCC patients, *n* = 523. Data from TCGA, PanCancer Atlas. **B** Bar graph showing *BIRC2* and *BIRC3* DNA amplification mutations in OSCC patients, *n* = 187. Patient DNA samples from OSCC Clinical Sample Bank of Nanjing Stomatological Hospital. **C** Comparison of the cIAP1 expression between OSCC patients with (n = 9)/without (n = 14) *BIRC2* amplification mutation. *P* = t test. **D** Comparison of the cIAP2 expression between OSCC patients with (n = 12)/without (n = 14) *BIRC3* amplification mutation. *P* = t test. **E** Representative IHC staining and graphical summary of cIAP1 in OSCC tissues from patients with (n = 9)/without (n = 14) *BIRC2* amplification mutation. **F** Representative IHC staining and graphical summary of cIAP2 in OSCC tissues from patients with (n = 12)/without *(n* = 14) *BIRC3* amplification mutation. Graphical depiction of overall survival (OS) (**G**), metastasis-free survival (MFS) (**H**), recurrence-free survival (RFS) (**I**), and disease-free survival (DFS) (**J**) from low transcription (*n* = 47) and high transcription mRNA levels (n = 41) of *BIRC2* in OSCC patients, *P* = Log-rank (Mantel–Cox) test. Graphical depiction of OS (**K**), MFS (**L**), RFS (**M**), and DFS (**N**) from low transcription (*n* = 41) and high transcription mRNA levels (*n* = 47) of *BIRC3* in OSCC patients, *P* = Log-rank (Mantel–Cox) test. Error bars are Mean ± SD.

Since SM-induced cell death relies on the ability to antagonize IAPs, we first evaluated DNA mutation, mRNA, and protein expression of *BIRC2* (encoding cIAP1) and *BIRC3* (encoding cIAP2) in the TCGA database [34, 35] and from our OSCC clinical sample bio-bank (Affiliated Stomatological Hospital, Medical School of Nanjing University) to determine whether the targets of SMs were aberrantly expressed in OSCC. Most of SMs such as birinapant [36], AT406 [37], LCL161 [38], ASTX660 [39], and AZD5582 [40] have higher affinity for cIAP1 and/or cIAP2 than XIAP, therefore we excluded XIAP expression analysis in this study. In the TCGA, PanCancer Atlas [34], we found that 5.35% and 5.74% of HNSCC patients had *BIRC2* or *BIRC3* amplification mutations respectively (Fig. 1A). In an additional pan-cancer dataset (ICGC, TCGA, Pan-cancer whole genome analysis, Nature 2020) [35], we found that HNSCC patients had the second highest *BIRC2* and *BIRC3* mutation frequency among 27 types of cancers with a prevalence of 14.29% (Supplementary Fig. 1A), similar to Eytan et al. [19]. To specifically obtain the mutation landscape in OSCC patients, we further analyzed the *BIRC2* and *BIRC3* amplification mutation rates in our OSCC bio-bank samples. We found that the amplification mutation of *BIRC2* and *BIRC3* was 4.81% (9/187) and 6.42% (12/187) (Fig. 1B), which was similar to the proportions in HNSCC patients. These data suggested that amplification mutation of *BIRC2* and *BIRC3* exists in HNSCC patients, including OSCC patients.

Moreover, to clarify the clinical relevance of the amplification mutations, we performed survival analysis of OSCC patients with/without *BIRC2/BIRC3* amplification mutation. In comparison to the survival curves of normal OSCC patients, patients bearing *BIRC2/BIRC3* amplification mutation had a tendency to have a worse prognosis (Supplementary Fig. 2A–D). But the number of cases involved was insufficient to achieve statistical significance. Furthermore, we collected these patients’ frozen tumor tissues from our biobank to prepare paraffin sections for Immunohistochemistry (IHC) for cIAP1/cIAP2. We observed that OSCC patients with *BIRC2/BIRC3* amplification mutation exhibited higher expression levels of cIAP1 (*P* = 0.0204) and cIAP2 (*P* = 0.0001) (Fig. 1C–F), which may be correlated to the poorer prognosis.

*BIRC2* and *BIRC3* mRNA levels in tumor and paired non-tumor tissues in HNSCC patients were further analyzed. The data was extracted from the TCGA database [34] and a comparative analysis revealed that *BIRC2* mRNA levels were significantly higher in tumor tissues compared to normal tissues (*P* = 0.0028) (Supplementary Fig. 2E). *BIRC3* mRNA levels were not significantly different between tumor and normal tissues (Supplementary Fig. 2F). Subsequently, cDNA samples of 88 OSCC patients were collected from our bio-bank and a prognostic value relating to *BIRC2* and *BIRC3* mRNA expression was analyzed. This analysis indicated that those with higher *BIRC2* transcriptional levels had poorer overall survival (OS) (*P* = 0.0126), but it did not influence metastasis-free survival (MFS), recurrence-free survival (RFS) or disease-free survival (DFS) (Fig. 1G–J). Additionally, *BIRC3* mRNA expression did not exhibit any significant influence on OS, MFS, RFS, and DFS (Fig. 1K–N). These findings suggest that a higher *BIRC2* transcriptional level contributed to OSCC progression.

**Fig. 2:**
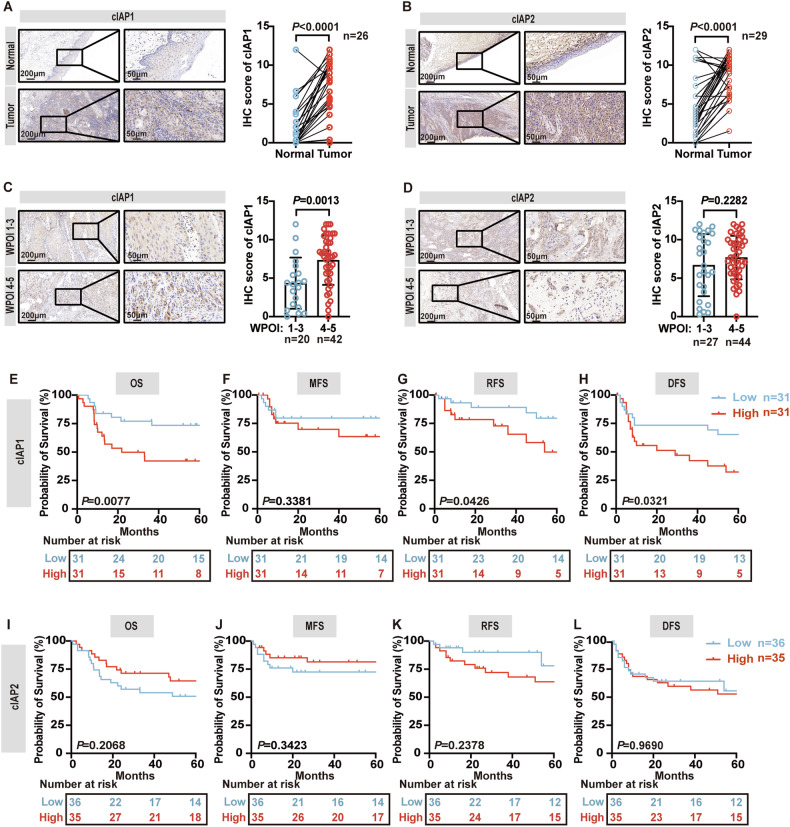
cIAP1 is aberrantly expressed in OSCC and predicts poor clinical outcomes. Representative IHC staining and graphical summary of cIAP1 (*n* = 26) (**A**) and cIAP2 (*n* = 29) (**B**) in OSCC tissues and normal epithelium tissues from the same patient, *P* = paired *t*-test. **C** Representative images and graphical summary of cIAP1 expression in OSCC patients with WPOI I-III (*n* = 20) and WPOI IV-V (*n* = 42), *P* = *t*-test. **D** Representative images and graphical summary of cIAP2 expression in OSCC patients with WPOI I-III (*n* = 27) and WPOI IV-V (*n* = 44). *P* = *t*-test. Graphical depiction of OS **(E)**, MFS (**F**), RFS (**G**), and DFS (**H**) for low (*n* = 31) and high expression levels (*n* = 31) for protein cIAP1, *P* = Log-rank (Mantel–Cox) test. Graphical depiction of OS (**I**), MFS (**J**), RFS (**K**), and DFS (**L**) for low (*n* = 36) and high expression levels (*n* = 35) for protein cIAP2, *P* = Log-rank (Mantel–Cox) test. Error bars are Mean ± SD.

IHC staining for cIAP1 and cIAP2 proteins was conducted to examine the expression profile of these proteins in OSCC. Consistent with the mRNA analysis (Supplementary Fig. 2E), cIAP1 protein expression was higher in OSCC compared to normal oral epithelium (*P* < 0.0001) (Fig. 2A). However, despite the absence of significant differences in *BIRC3* mRNA levels between tumor areas and normal epithelium in the TCGA database (*P* < 0.0001), high expression levels of cIAP2 were observed specifically in OSCC tumor areas compared to normal epithelium (*P* < 0.0001) (Fig. 2B, Supplementary Fig. 2F). Basing on the tumor tissue IHC score, patients were divided into low and high cIAP1 /cIAP2 expression groups (Supplementary Fig. 3A, B). A chi-squared test was employed to analyze the diagnostic values of cIAP1 and cIAP2, which demonstrated that higher expression of cIAP1 was correlated with a higher mortality risk (*P* = 0.01), inferior differentiation (*P* = 0.038) and a higher worst pattern of invasion (WPOI) pathology grade (*P* = 0.001) (Supplementary Table 1).

A positive correlation was observed between higher expression of cIAP2 and poorer differentiation (*P* = 0.048) (Supplementary Table 2). Comparison of the expression levels of cIAP1 and cIAP2 in OSCC patients with WPOI I-III and WPOI IV-V revealed that patients with WPOI IV-V exhibited a higher expression of cIAP1 than those with WPOI IV-V (*P* = 0.0013) (Fig. 2C), whereas there was no significant difference in cIAP2 expression (Fig. 2D). In addition, Kaplan–Meier survival curve analysis indicated that patients with high cIAP1 expression levels exhibited reduced OS (*P* = 0.0077), RFS (*P* = 0.0426) and DFS (*P* = 0.0321) (Fig. 2E–H), while there was no significant correlation between cIAP2 expression and survival (Fig. 2I–L). Furthermore, multivariate Cox regression analysis adjusted for established clinical risk factors (eg. age at diagnosis, TNM stage, pathological stage, local recurrence, and distant metastasis) indicated that lymph node metastasis (HR = 3.31, *P* = 0.009), distant metastasis (HR = 2.802, *P* = 0.013) and high expression of cIAP1 (HR = 2.608, *P* = 0.035) were risk factors for OS in OSCC (Supplementary Table 3). Notably, high cIAP1 expression was identified as an independent prognostic factor for OSCC, which was not the case for cIAP2 (Supplementary Tables 3, 4). In conclusion, elevated cIAP1 expression was a predictor of a poor clinical outcome in OSCC.

### OSCC tumor cells exhibit differential sensitivity to SM treatments

**Fig. 3:**
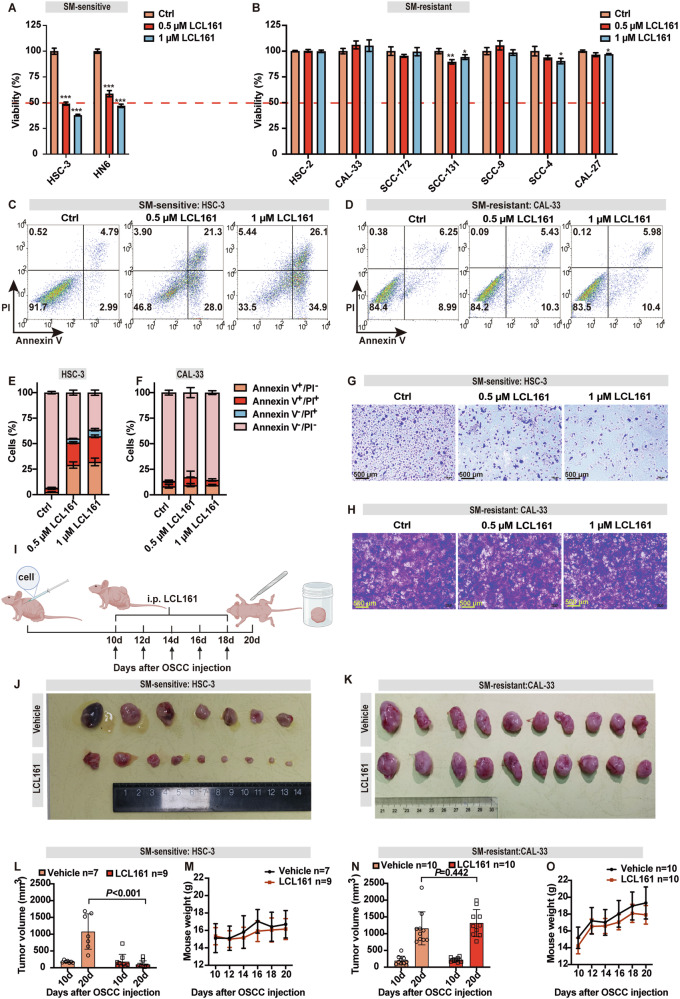
OSCC tumor cells exhibit variable sensitivity to SM. Cell viability measured by CCK-8 of OSCC sensitive (**A**) or resistant (**B**) cell lines after treatment with SM LCL161 (0.5 or 1 μM) for 24 h, * *P* < 0.05, ** *P* < 0.01, *** *P* < 0.001, *P* = ANOVA analysis. Representative dot plots of the proportion of live (AV^−^/PI^−^), early apoptotic (AV^+^/PI^−^), and late apoptotic (AV^+^/PI^+^) in HSC-3 (**C**) and CAL-33 cells (**D**) post-treatment with SM LCL161 for 24 h. Bar graphs showing the percentage of HSC-3 (**E**) and CAL-33 cells (**F**) within each quadrant. Representative images showing crystal violet staining of HSC-3 (**G**) and CAL-33 (**H**) cells post-treatment for 24 h with/without 0.5 or 1 μM LCL161. **I** Diagrammatic summary and timeline of the experimental protocol for the transplantation of OSCC cells into nude mice subcutaneously and treatment with vehicle or LCL161 (via intraperitoneal injection). Representative images of gross tumor lesions obtained from nude mice bearing with HSC-3 (**J**) or CAL-33 cell (**K**). Graphical representation of tumor volume (**L**, **N**) and mouse weight (**M**, **O**) from nude mice subcutaneously transplanted with HSC-3 or CAL-33 cells, *P* = *t*-test. Error bars are Mean ± SD.

Given the correlation between high expression of cIAP1 and poor prognosis in OSCC, we tested the efficacy of SMs in OSCC. OSCC cell lines were treated with monovalent SMs LCL161 or AT406. As observed in other tumor types [41], OSCC cell lines exhibited differential sensitivity to SM treatment (Fig. 3A, B, Supplementary Fig. 4A, B). LCL161 was selected for further experiments due to its slightly superior efficacy compared to AT406 (Fig. 3A, B, Supplementary Fig. 4A, B). OSCC cell lines HSC-3 and HN6 were sensitive (Fig. 3A) and CAL-33, HSC-2, SCC-172, SCC-131, SCC-9, SCC-4 and CAL-27 were resistant (Fig. 3B) to LCL161 killing. Additionally, we found LCL161 was unable to induce human epidermal keratinocyte cell line (HACAT) death (Supplementary Fig. 4C). SM-sensitive HSC-3 and HN6, and SM-resistant CAL-33, CAL-27, and SCC-9 cell lines were chosen as representatives for the majority of the following experiments in this study. Moreover, flow cytometry analysis using Annexin-V FITC/PI demonstrated that a higher proportion of HSC-3 (Fig. 3C, E) and HN6 (Supplementary Fig. 4D, E) cells underwent early (AV^+^/PI^−^) and late apoptosis (AV^+^/PI^+^) compared to CAL-33 (Fig. 3D, F) following 24 h of LCL161 treatment. Mirroring the survival assays, crystal violet clonogenic survival assays showed that HSC-3 and HN6 cells were effectively killed by LCL161 treatment while few CAL-33 cells died (Fig. 3G, H, Supplementary Fig. 4F).

To verify the efficiency of LCL161 in vivo, 4-week-old BALB/c nude female nude mice (GemPharmatech, China) were used to construct a subcutaneous OSCC xenograft model. 2 × 10^6^ HSC-3 or CAL-33 cells were inoculated subcutaneously into the right armpit. (Fig. 3I). The progression of the HSC-3 subcutaneous xenograft model treated with LCL161 was significantly blunted in comparison to the vehicle group (Fig. 3J, L). In contrast, LCL161 treatment failed to reduce tumor growth in mice bearing CAL-33 xenografts, which is consistent with the in vitro data (Fig. 3K, N). These results indicated that OSCC tumor cell lines display differential sensitivities to SM treatment. Additionally, there was no difference in tumor recipient mouse body weight between the LCL161 treatment group and vehicle group (Fig. 3M, O). We subsequently performed hematoxylin and eosin (H&E) staining of the recipient mouse livers and spleens. We did not observe alterations in the pathologic features associated with drug injury, such as vacuolar degeneration of hepatocytes and enrichment of splenic lymphocytes in LCL161 treatment group. There was some indication of splenomegaly and disorganization of lymphoid follicles in the spleen after LCL161 treatment however this is consistent with the ability of SMs to activate non-canonical NF-κB (Supplementary Fig. 4G). These results demonstrate the dosing safety profile of LCL161 in vivo treatment.

### cIAP1 is degraded upon SM treatment in both OSCC-sensitive and resistant tumor cells

**Fig. 4:**
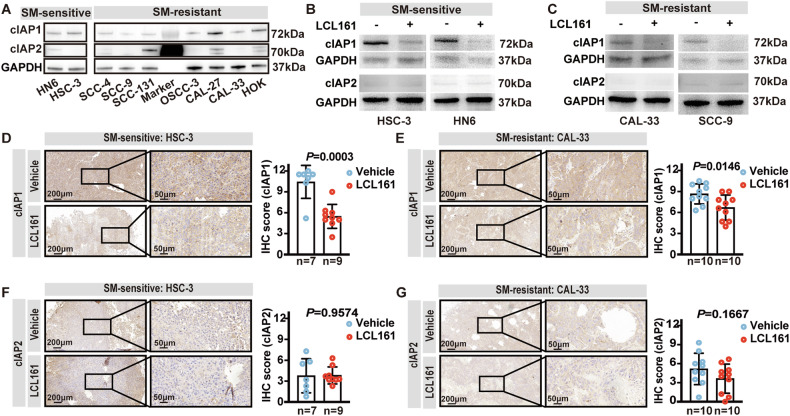
SM induces cIAP1 degradation in both SM-sensitive and resistant OSCC tumor cells. **A** Western blot showing original expression levels of cIAP1 and cIAP2 for the indicated cell lines without any stimulation. Western blot showing expression levels of cIAP1 and cIAP2 pre and post-treatment with/without LCL161 1 μM (+) in SM-sensitive cell lines (**B**) and SM-resistant cell lines (**C**) for 24 h. Representative IHC images of cIAP1 (**D**) and cIAP2 (**F**) and graphical quantitation from tumor slides obtained from HSC-3 bearing mice, *P* = Student’s *t*-test. Representative IHC staining of cIAP1 (**E**) and cIAP2 (**G**) and graphical quantitation of tumor slides obtained from CAL-33 bearing mice, *P* = Student’s *t*-test. Error bars are Mean ± SD.

Since OSCC cells showed differential responses to SM treatment, we sought to analyze the factors that might contribute to SM therapy resistance. First, the expression of cIAP1 and cIAP2 was assessed by western blot (WB). We found that both sensitive and resistant OSCC cell lines expressed cIAP1 pre SMs stimulation, but the expression levels did not obviously correlate with their sensitivity to SM therapy (Fig. 4A). Compared to cIAP1, cIAP2 expression was more variable, but again did not correlate with SM sensitivity (Fig. 4A). In summary, the ability of SM therapy to induce OSCC death did not appear to correlate with cIAP1 or cIAP2 expression levels.

Next, we assessed SM-induced degradation of cIAP1 and cIAP2 in OSCC cells. In this instance, cIAP1 levels were decreased after treatment with LCL161 for 24 h regardless of whether the cells were SM-sensitive or not (Fig. 4B, C). In comparison, cIAP2 expression remained low and unaltered (Fig. 4B, C). To determine whether SM treatment elicited a similar pharmacological response in vivo, we performed IHC staining for cIAP1 and cIAP2 in BALB/c nude mice subcutaneously grafted with HSC-3 or CAL-33 cells and treated with LCL161. Consistent with our in vitro data, both HSC-3 and CAL-33 transplanted mice showed reduced expression levels of cIAP1 (*P* = 0.0003, *P* = 0.0146) (Fig. 4D, E) but not cIAP2 after treatment with LCL161 (Fig. 4F, G). These results demonstrated that LCL161 successfully induced cIAP1 degradation in both the HSC-3 and CAL-33 cell xenograft models after treatment with LCL161.

### Addition of exogenous TNF reverses SM resistance in a subset of OSCC cells

**Fig. 5:**
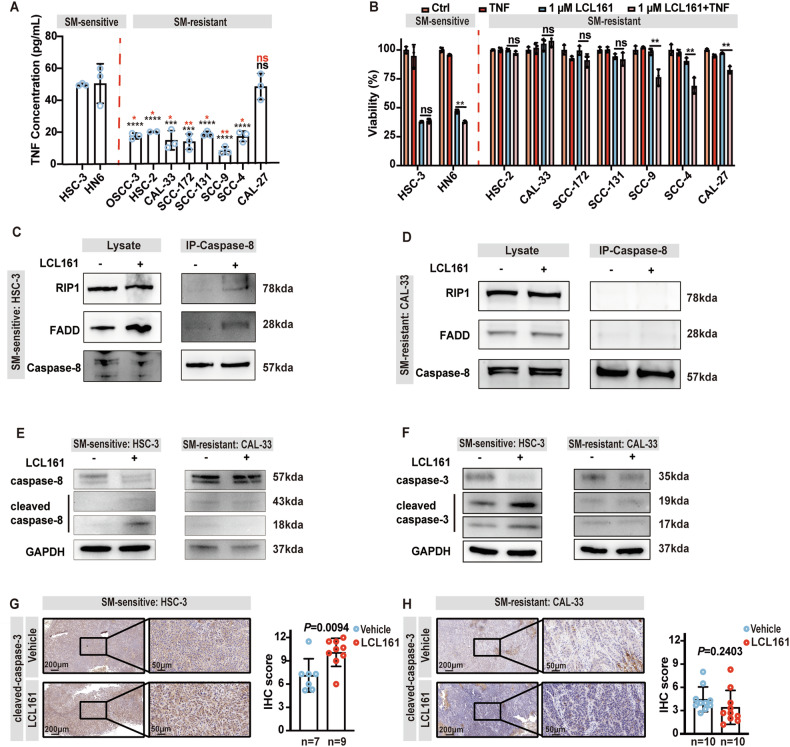
OSCC-resistant cells fail to form Complex IIa. **A** Graphical representation of Elisa autocrine TNF measurements from nine OSCC cell lines. * *P* < 0.05, ** *P* < 0.01, *** *P* < 0.001, *** *P* < 0.0001, *P* = ANOVA analysis. Compared with the TNF concentration of HSC-3: mark ‘*’ in black, compared with the TNF concentration of HN6: mark ‘*’ in red. **B** Graphical representation of the percentage viability of OSCC cell lines following treatment with LCL161 combined with TNF (10 ng/mL), * *P* < 0.05, ** *P* < 0.01, *** *P* < 0.001, *P* = ANOVA analysis. Assessment of Complex IIa formation by immunoprecipitation for RIP1, FADD, and caspase-8 in HSC-3 (**C**) and CAL-33 (**D**) after LCL161 treatment. Western blot detection for the cleavage of caspase-8 (**E**) and caspase-3 (**F**) in HSC-3 and CAL-33 after LCL161 stimulation. Representative images and quantitative analysis of IHC staining for cleaved-caspase-3 from tumors isolated from HSC-3 (**G**) or CAL-33 (**H**) bearing nude mice. *P* = Student’s *t*-test. Error bars are Mean ± SD.

SMs induce cell death as a single agent by simultaneously inducing autocrine TNF production and sensitizing cells to TNF killing [13, 42]. We therefore measured the TNF concentrations in both SM-sensitive and resistant OSCC cells using ELISA. Surprisingly, all tested cells had detectable basal TNF expression (Fig. 5A) in the absence of any stimulation. However, the SM-sensitive cells secreted a comparatively higher concentration of TNF compared to that of SM-resistant cells, excepting CAL-27 cells (Fig. 5A). Since SMs-induced cell death is correlated with TNF concentration [43, 44], we also tested the TNF concentration after LCL161 treatment via ELISA. Interestingly, we only observed a significant upregulation of autocrine TNF in SM-sensitive HSC-3 and HN6 cells after 24 h, although approximately 50% of the cells were apoptotic at this time (Supplementary Fig. 5A, Fig. 3A).

Previous studies have reported that resistance to SM therapy can be overcome by upregulation of TNF either directly or indirectly [24, 45, 46]. To exclude the possibility that insufficient TNF leads to SM resistance, we treated all OSCC cells with or without high concentrations of exogenous TNF plus LCL161. However, while the addition of TNF had a minor effect in reversing LCL161 resistance in SCC-9, SCC-4, and CAL-27 cells, it had no effect on the four other OSCC cell lines (Fig. 5B). Therefore, while TNF was important for promoting SM-mediated cell death, it did not appear to be the key factor conferring resistance to SM treatment in OSCC cells.

### OSCC-resistant cells fail to form Complex IIa after SM treatment

Since we found that both resistant and sensitive cells produce TNF, we examined whether they assembled Complex IIa in response to SM treatment. Consistent with the death data, LCL161 induced a RIPK1-caspase-8 containing complex in SM-sensitive HSC-3 but not CAL-33 cells (Fig. 5C, D). Cleavage of caspase-8 and cleavage of caspase-3 are indicators of SM-induced activation of apoptotic pathway [13, 29], and consistently, SM treatment promoted the increased cleavage of total caspase-8 and caspase-3 protein in SM-sensitive HSC-3 cells compared to SM-resistant CAL-33 cells (Fig. 5E, F). We also sought to verify this finding in vivo by staining for cleaved-caspase-3 in our subcutaneous xenograft models. Consistent with our previous results, cleavage of caspase-3 was only observed in SM-sensitive HSC-3 subcutaneous xenograft mice treated with LCL161 (Fig. 5G) but not in mice bearing SM-resistant CAL-33 (Fig. 5H).

### SMs activate the non-canonical NF-κB pathway in both SM-sensitive and resistant OSCC cells

**Fig. 6:**
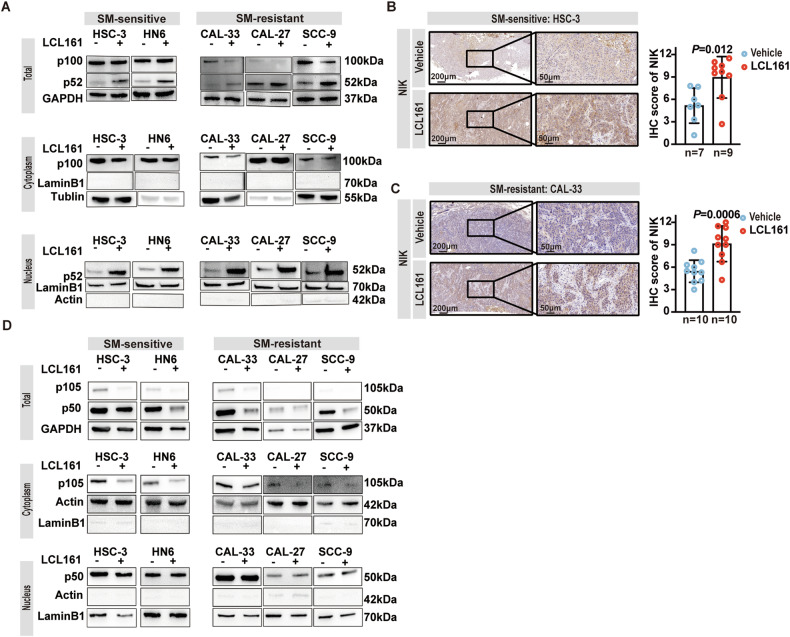
SM activates non-canonical NF-κB pathway in both SM-sensitive and resistant OSCC cells. **A** Representative WB of total, cytoplasmic, and nuclear fractions of NF-κB2 (p100 and p52) in HSC-3, HN6, CAL-33, CAL-27, and SCC-9 OSCC cell lines treated with LCL161 for 24 hours. Representative images of IHC staining for NIK protein and quantitative analysis from tumor tissue isolated from HSC-3 (**B**) or CAL-33 (**C**) bearing nude mice treated with Vehicle or LCL161, *P* = Student’s *t*-test. Error bars are Mean ± SD. **D** Representative western blots of total, cytoplasmic, and nuclear fractions of NF-κB1 (p105 and p50) in HSC-3, HN6, CAL-33, CAL-27, and SCC-9 OSCC cell lines after 24 h treatment with LCL161.

In addition to modulating cell death, SMs affect cell survival via opposing effects on the canonical (NF-κB1/RelA) and non-canonical (NF-κB2/RelB) NF-κB pathways [13]. Specifically, SMs prevent TNF-induced activation of the canonical NF-κB pathway and divert the pro-survival complex to a death complex. On the other hand, SMs activate the non-canonical NF-κB pathway by inhibiting cIAP-induced degradation of the non-canonical NF-κB activating kinase (NIK) [47–49]. To determine whether activation of non-canonical NF-κB might contribute to resistance, we examined the activation of non-canonical NF-κB in 5 OSCC cell lines after LCL161 treatment for 24 h. Consistent with previous reports [50], nuclear-localized p52 (NF-κB2) levels were significantly increased (Fig. 6A). Consistent with the in vitro studies, both SM-sensitive HSC-3 and SM-resistant CAL-33 xenografts showed higher IHC score of NIK after LCL161 injection (Fig. 6B, C). We then investigated NF-κB1 activation, although cytoplasmic localized unprocessed p105 expression levels, which were downregulated, with no significant change in nuclear p50 (NF-κB1) observed in each of the 5 OSCC cell lines after treatment with LCL161 for 24 h (Fig. 6D).

### ABCA3, ABCB1, and ABCB4 upregulation in OSCC cells contributes to SM resistance

**Fig. 7:**
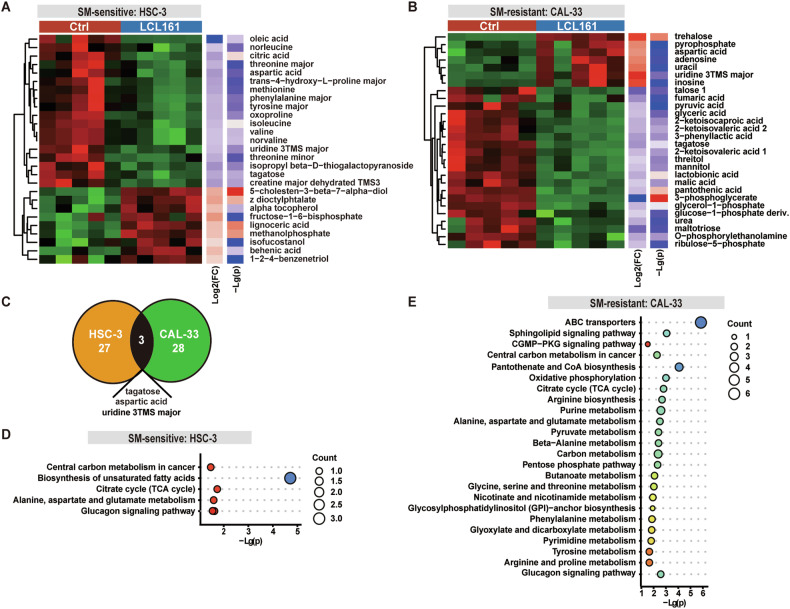
SM-resistant cells exhibited enrichment of the ABC transporter pathway. Heatmaps showing differential metabolite expression (variable importance for projection >1, *P* < 0.05) in HSC-3 (**A**) and CAL-33 (**B**) OSCC cell lines post control (Ctrl) or treatment with 1 μM LCL161 for 6 h. **C** Venn diagram showing the numbers of overlapping and differentially expressed metabolites in HSC-3 and CAL-33 OSCC cell lines post LCL161 treatment. KEGG pathway enrichment analysis of differential metabolites for HSC-3 (**D**) and CAL-33 (**E**) cells post LCL161 treatment for 6 h.

An increasing number of studies have suggested that the IAP family regulates cellular metabolism [51, 52]. After excluding well-known factors (cIAPs levels, TNF concentration, and NF-κB pathway activation) as determinants of the differential sensitivity of OSCC cells to SM treatment, we performed gas chromatography-mass spectrometry (GC-MS) to identify metabolic differences between SM-sensitive and resistant cells after SM stimulation for 6 h. A total of 27 metabolites were found to be significantly differentially expressed between sensitive HSC-3 and HSC-3 plus LCL161 (Fig. 7A), while 28 metabolites were found to be significantly different between resistant CAL-33 and CAL-33 plus LCL161 (Fig. 7B). Only 3 metabolites overlapped: tagatose, aspartic acid, and uridine 3TMS major, indicating that the HSC-3 treated group didn’t share a similar metabolic trend with the CAL-33 treated group (Fig. 7C). Moreover, the basal metabolites of HSC-3 and CAL-33 were undoubtedly different (Supplementary Fig. 6A).

**Fig. 8:**
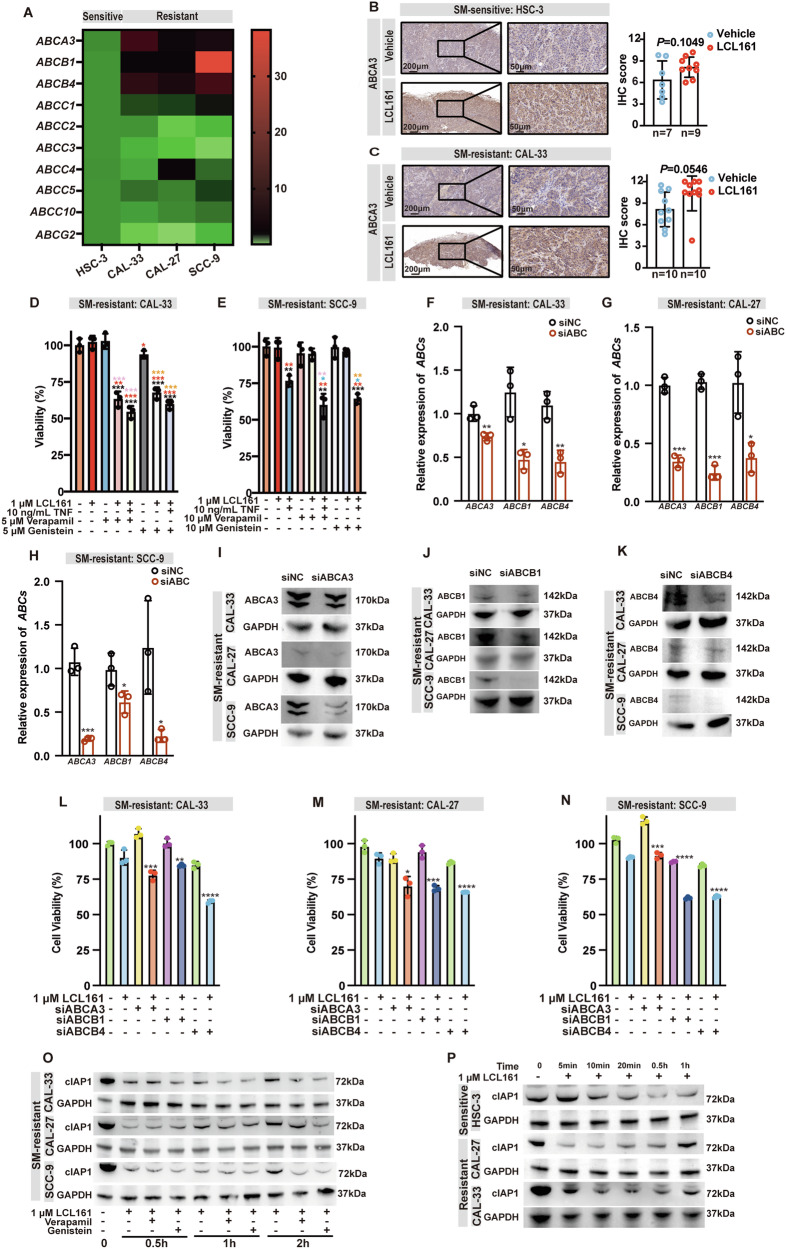
Upregulation of the ABC transporter proteins ABCA3, ABCB1, and ABCB4 in OSCC cells contributes to SM resistance. **A** Heatmap of showing basal transcription levels of the ABC transporter families from CAL-33, CAL-27, and SCC-9 OSCC cells, with HSC-3 used as control. Representative images of IHC staining for ABCA3 protein and quantitative analysis from tumor tissue isolated from HSC-3 (**B**) or CAL-33 (**C**) subcutaneous xenograft tumor model treated with Vehicle or LCL161, *P* = Student’s *t*-test. Graphical representation of the viability percentage of CAL-33 (**D**) and SCC-9 (**E**) OSCC cell lines following the indicated treatments. * *P* < 0.05, ** *P* < 0.01, *** *P* < 0.001. *P* = ANOVA analysis. Compared with the control group: mark ‘*’ in black, compared with the group treated with 1 μM LCL161: mark ‘*’ in red, compared with the group treated with 1 μM LCL161 and 10 ng/mL TNF: mark ‘*’ in blue, compared with the group treated with verapamil: mark ‘*’ in pink, compared with the group treated with genistein: mark ‘*’ in yellow. mRNA expression of *ABCA3, ABCB1* and *ABCB4* from SM-sensitive OSCC cell lines CAL-33 (**F**), CAL-27 (**G**) and SCC-9 (**H**) after knockdown with siRNA. Protein expression of ABCA3 (**I**), ABCB1 (**J**), and ABCB4 (**K**) from SM-sensitive OSCC cell lines CAL-33, CAL-27, and SCC-9 after knockdown with siRNA. Graphical representation of the viability percentage of CAL-33 (**L**), CAL-27 (**M**), SCC-9 (**N**) OSCC cell lines with siABCA3/siABCB1/siABCB4 following the indicated treatments. Compared with the relative controls: * *P* < 0.05, ** *P* < 0.01, *** *P* < 0.001. *P* = ANOVA analysis. **O** Representative western blots of cIAP1 expression of CAL-33, CAL-27, and SCC-9 treated with LCL161 combined with genistein (5 μM) or verapamil (5 μM) for 0-2 h. **P** Representative western blots of cIAP1 expression of HSC-3, CAL-33, and CAL-27 treated with LCL161 for 0–1 h. Error bars are Mean ± SD.

A KEGG pathway enrichment analysis found that LCL161 treatment was associated with a signature for unsaturated fatty acids biosynthesis in HSC-3 (Fig. 7D), whereas this treatment resulted in a signature for ABC transporter pathway activation in CAL-33 (Fig. 7E). Although the mRNA levels of several ABC transporter family members were not significantly increased in the SM-resistant CAL-33, CAL-27, and SCC-9 cell lines, after LCL161 treatment (Supplementary Fig. 7A-E), mRNA levels of ABCA3, ABCB1, and ABCB4 were higher in the SM-resistant cells than in the sensitive HSC-3 in the absence of any stimulation (Fig. 8A). The upregulation of ABCA3 in CAL-33 subcutaneous xenografts (*P* = 0.0546) was more pronounced than in HSC-3 subcutaneous xenografts (*P* = 0.1094) after LCL161 injection (Fig. 8B, C), but it was not statistically significant, which was consistent with the results of mRNA expression analysis (Supplementary Fig. 7A, C).

Based on these findings, we speculated that SM-resistant OSCC cells might efflux SMs to gain resistance. Therefore, in addition to SM treatment, we also inhibited ABC transporters using the ABCA3 inhibitor, genistein [53], and the ABCB1 and ABCB4 inhibitor, verapamil [54–56] and evaluated their ability to synergistically induce cell death in OSCC cell lines. Consistent with the idea that ABC transporters are more active in resistant cells, LCL161 in combination with ABC inhibitors induced more cell death than LCL161 alone in SM-resistant CAL-33(Fig. 8D). For SCC-9, the addition of exogenous TNF makes this combination regimen effective (Fig. 8E). To further validate the hypothesis, we subsequently targeted knockdown of ABCA3, ABCB1and ABCB4 using siRNA (Fig. 8F-K) and found that SM-resistant cell lines after silencing *ABCA3*, *ABCB1* and *ABCB4* were more sensitive to SM-induced death (Fig. 8L-N). Supporting the idea that LCL161 is an ABC transporter substrate that is actively transported out of the cell. Consistently, we also observed that LCL161, in combination with ABC transporter inhibitors, induced more complete degradation of cIAP1 in SM-resistant cell lines (Fig. 8O), but did not influence SM-sensitive cell lines (Supplementary Fig. 8A). In addition, we compared the extent of cIAP1 degradation in SM-sensitive and resistant cells and found that although there was no significant difference in cIAP1 degradation in the two sub-type cell lines after 24 h of LCL161 stimulation (Fig. 4B, C), resistant cells treated with LCL161 for a short time (0–1 h) showed inexhaustive degradation of cIAP1 and even a gradual increase in the expression of cIAP1, while the sensitive cells displayed decreased expression of cIAP1 (Fig. 8P). The degradation of cIAP1 by SMs, particularly during the first few hours of treatment, appeared to correlate with OSCC sensitivity to SMs.

## Discussion

While SMs have been proven to be safe, even in combination with other cytotoxic drugs, they have not until recently, dramatically improved treatment outcomes [13]. In recent trials where the SM xevinapant was combined with standard-of-care chemo-radiotherapy in HNSCC, dramatic improvements were observed: the risk of death or disease progression was reduced by 67% [32, 33]. These results provide a strong impetus to discover why HNSCCs, of which OSCCs are an important subset, respond to SM treatment.

We considered that IAP expression levels might correlate with sensitivity to SMs [57, 58], and therefore assessed IAP expression levels in OSCC. Approximately 5–9% of OSCCs contain amplifications of *BIRC2* (encoding cIAP1) and *BIRC3* (encoding cIAP2) locus 11q22.1-11q22.2, both in the TCGA database and from 88 local patients at the DNA level. Note that the BIRC2 and BIRC3 copy number we measured here are not Copy Number Variations (CNVs). They only predict an increased number of copies, and there may be false positives and false negatives. The expression level of cIAP2 protein was also higher in tumors. However, comparison of cIAP2 mRNA levels between tumor and normal counterparts were not significantly different in the TCGA cohort. This discrepancy may be due to different patient cohorts being assessed. In the present study, we were unable to obtain mRNA from the OSCC patient cohort, therefore we used an IHC approach to measure protein expression levels. Further studies assessing cIAP2 mRNA and protein levels in OSCC are required to confirm if this discrepancy was due to the TCGA cohort containing HNSCC samples rather than wholly OSCC. High transcription level of *BIRC2* was predictive of poorer patient outcomes, as was higher cIAP1 protein expression in tumors, compared to paired non-tumor counterparts. However, high cIAP2 was actually predictive of a better outcome. cIAP2 expression is increased upon SM treatment most likely as a result of non-canonical NF-κB activation caused by cIAP1 degradation [59]. Therefore, it is plausible that high levels of cIAP2 indicate low levels of cIAP1 and that these two observations are consistent with each other. While the SMs we used in our study target cIAP1(*BIRC2*), cIAP2 (*BIRC3*) and XIAP (*BIRC4*), we did not examine XIAP levels in our resistant and sensitive cell lines and it, therefore, remains possible that XIAP levels could affect sensitivity. Nevertheless, mutations affecting XIAP or caspase-9 occur rarely in HNSCC compared to cIAP1/2 [60, 61] and extrinsic apoptosis effectors such as FADD and caspase-8, suggesting that XIAP and the intrinsic pathway regulated by it are not as important in HNSCC.

TNF is required for SM mediated cell death [14, 15], and overall we observed a correlation between sensitivity to SM and TNF expression, even though one of the seven resistant cell lines expressed TNF to similar levels as the two sensitive cell lines. While the transcription factor specificity protein 3 (SP3) has been shown to confer differential sensitivity to SM by regulating TNF expression [62]. However, TNF production was not the only limiting factor, since addition of exogenous TNF only partially reversed resistance to SM treatment in three of the resistant cell lines. In addition, all OSCC cell lines tested secreted TNF, and while the two sensitive lines secreted the highest levels of TNF, one of the resistant lines secreted very similar amounts as these two. However, only sensitive cells increased secretion of TNF upon SM treatment by twofold or more. Notably, non-canonical NF-κB can contribute to TNF production and this pathway was also activated in both SM-sensitive and resistant cells. The SIX transcriptional repressors have also been shown to suppress SM mediated death [63], by inhibiting non-canonical NF-κB activation through NF-κB responsive promoters. Therefore, it is possible that differences in SIX expressions could influence and inhibit SM-induced death post p100 processing and p52 translocation into the nucleus.

The ABC transporter family is responsible for multi-drug resistance through drug efflux [64]. Clinical ABCB1 inhibitors enhance the bioavailability of a number of different SMs [65], and we have shown that LCL161 is a direct substrate of ABCB1 [66]. In this study, we found that SM-sensitive cells had uniformly low levels of *ABC* family members while resistant cells had high levels of *ABCA3, ABCB1,* and *ABCB4*. These seem to contribute to LCL161 resistance since co-treatment with verapamil (an ABCB1 and ABCB4 inhibitor) or genistein (an ABCA3 inhibitor) protected them from apoptosis. Currently, ABC transporter inhibitors have not been successfully deployed in the clinic to overcome multidrug resistance. However, they have usually been combined with cytotoxic drugs, rather than drugs like SMs, which are considerably less toxic to healthy cells [67]. Indeed the SM, ABC transporter inhibitor combination was well tolerated and efficacious in treating animal models of infection and leukemia [44, 65]. Furthermore, the ABC transporter inhibitor, lazertinib was found to improve the efficacy of chemotherapy in patients with ABCB1 or ABCG2 overexpression [68]. Therefore, the combination of SMs and lazertinib could have promising therapeutic potential for OSCC patients with high ABC transporter expression.

In summary, our study fills a gap in knowledge relating to how SM drug resistance arises and potentially how it can be overcome to improve outcomes for OSCC. We assessed a considerable number of clinical OSCC samples to analyze the correlation between cIAP expression and OSCC patients’ prognosis. Our clinical sample data indicates that patients with OSCC that overexpress cIAP1 have lower survival rates, suggesting cIAP1 is a valid therapeutic target, especially for SMs that promote cIAP1 destruction. However, degradation of cIAP1 is associated with a number of outcomes, including non-canonical NF-κB activation and, in some cells, TNF production which can either combine to kill cells or in some cases make them more resistant to cell death. Thus, for example, in lymphomas, activation of non-canonical NF-κB is a strong survival signal, and therefore mice with Myc lymphomas treated with LCL161 have decreased survival [69]. Furthermore, while for some cells SM induced autocrine production of TNF and loss of cIAP1 is sufficient to induce apoptosis [14, 15], our data show that for a subset of OSCC, even the addition of exogenous TNF is not sufficient to induce death in resistant cells. This is despite the fact that these resistant OSCC cells nevertheless express many of the extrinsic apoptosis effector molecules, including RIP1 FADD, caspase-8, and -3. However, the functional expression of these proteins was undetermined and more proteins have been reported to regulate formation or activity of the death-inducting signaling complex. Therefore, the basis of their resistance is still unclear. Finally, some, but not all SMs, are substrates of ABC transporters providing another route to resistance. This is particularly relevant to cancer cells that have already been subjected to cytotoxic therapy as resistance to some cytotoxic therapies can be driven by upregulation of ABC transporters [70]. Some of these resistance mechanisms can be accommodated, particularly if they’re known in advance. Nevertheless, in all likelihood SMs will, as in the recent HNSCC trials, be combined with chemo and or radiotherapy which may provide some of the necessary signaling to overcome resistance by, for example, upregulating TNF production and driving extrinsic apoptotic signaling independently of TNF [71]. Thus our findings may help improve outcomes for OSCC patients and other sub-groups of HNSCC [34] and potentially other solid cancers with elevated cIAPs, such as cervical, ovarian and bladder [19].

## Materials and methods

### Human ethics and sample information

All research complied with the relevant ethical regulations. OSCC patient samples and information were acquired with patient informed consent and handled within the guidelines and policies of the Research Ethics Committee of Nanjing Stomatology Hospital (NJSH-2021NL-026).

A total of 62 patients were included for cIAP1 IHC staining, and 71 patients were included for cIAP2 IHC staining. Tumor tissues and corresponding normal tissues were collected from 187 patients for DNA amplification mutation analysis. 88 of these samples were used for transcriptional levels analysis of *BIRC2* and *BIRC3*.

The inclusion criteria were: ① OSCC diagnosed by postoperative pathology; ② primary case; ③ no other treatment before surgery; ④ complete information on pathology and clinical follow-up. The exclusion criteria were: ① history of other malignant tumors; ② death due to reasons unrelated to OSCC. All samples and information were obtained from Affiliated Stomatological Hospital, Medical School of Nanjing University.

### Mice

4-week-old female BALB/c nude mice were purchased from GemPharmatech, Jiangsu, China. All mice were housed as follows: 12 light/12 dark cycles; temperature 28 °C, humidity 40–50%. All animal experiments were performed in accordance with the Jiangsu Association for Laboratory Animal Science protocols and subjected to review by the animal welfare and ethical review board of Nanjing University (IACUC-D2103002).

### Reagents and antibodies

Smac mimetics (LCL161, AT406) and ABC transporter inhibitors (genistein, verapamil) were purchased from Selleck, Shanghai, China. Additional information relating to the antibodies is listed in Supplementary Table 5.

### Cell culture

The cell lines used in this study were purchased from ATCC. All the cells were professionally verified to exclude mycoplasma or other contaminants. All cells were cultured in high glucose, Dulbecco’s Modified Eagle Medium (Thermo Fisher Scientific Inc, Massachusetts, USA) supplemented with 10% fetal bovine serum (Bio-Channel, Jiangsu, China) and 1% penicillin-streptomycin (New Cell and Molecular Biotech, Jiangsu, China). Cells were incubated at 37 °C in a standard humidified atmosphere of 5% CO2.

### DNA mutation analysis in TCGA

The cBIOPortal website (http://www.cbioportal.org/) for cancer genomics was used for the analysis of BIRC2 and BIRC3 gene mutations. These levels are derived from copy-number analysis algorithms like GISTIC or RAE. Copy number analysis indicates copy-number level per gene, in which “−2” is a deep loss (homozygous deletion), “−1” is a shallow loss (heterozygous deletion), “0” is diploid, “1” indicates a low-level gain, and “2” is a high-level amplification [34].

### DNA copy number analysis by qPCR

Copy number analyses of *BIRC2* and *BIRC3* were quantified by qPCR using the ViiA™ 7 Real-Time PCR System (Thermo Fisher Scientific Inc, Massachusetts, USA). *Globin* was used as the single-copy reference control and samples were assessed in triplicate. Primer sequences are listed in Supplementary Table 2. The SYBR Green Premix Pro Taq HS qPCR Kit (Accurate Biology, Hunan, China) was used according to the manufacturer’s instructions. PCR conditions: Step 1, 95 °C for 30 s for one cycle; Step 2, 95 °C for 5 s, 55 °C for 30 s, 72 °C for 30 s, repeated for 40 cycles; Step 3, dissociation. Copy number analysis of *BIRC2* and *BIRC3* was carried out using the comparative Ct method after validating that the efficiencies of PCR reactions of reference controls and genes under investigation are equal. Greater than 4 times gene copy number compared to reference control was deemed indicative of gene amplification.

### Mouse subcutaneous xenograft OSCC model

2 × 10^6^ HSC-3 or CAL-33 cells were inoculated subcutaneously into the right armpit. 10 days post-inoculation, when the tumor volume was about 150 mm^3^ (Tumor volume = length × width × width/2), mice were divided into Vehicle group or SM (LCL161) treatment group randomly. Each group had a minimum of at least 10 mice and average initial tumor size between groups was similar. LCL161 was prepared by dissolving in 0.1 N hydrochloric acid, followed by dilution in 0.1 N anhydrous sodium acetate (Solarbio, Beijing, China) to 70% of the total volume. Vehicle (30% 0.1 N hydrochloric acid + 70% 0.1 N anhydrous sodium acetate solution) or LCL161 (20 mg/kg) were injected intraperitoneally every two days, with five repeats. Body weight and tumor volumes were recorded during the experiment. Once the tumor volume exceeded 1500 mm^3^, or the tumor surface skin broke down, or the mice lost more than 20% of body weight, the mice were withdrawn from the experiment and were euthanized immediately. On the 20th day post OSCC inoculation, mice were euthanized and tumor tissues were collected. During the process of this experiment, no blinding was done.

### Cell viability assay: CCK-8

OSCC cell lines were dispersed into 96-well plates at 5000 cells per well, cultured overnight, followed by treatment with 0, 0.5 μM or 1 μM LCL161, or 8 μM AT406 combined with/without 10 ng/mL TNF (Peprotech, Massachusetts, USA) for 24 hours. Media was then supplemented with a 10:1 ratio of CCK-8 solution (Vazyme, Jiangsu, China) followed by culture for an additional 1–2 h. Absorbance values were read on a microplate reader (Molecular Devices, California, USA) at 450 nm wavelength. Plate reads were recorded when the control group absorbance value reached 0.8–1.2. Cell viability was calculated using the formula [(A-C)/(B-C)] × 100%, where A = experimental, B = control, C = blank (absorbance value of media and CCK-8 Solution only, without cells).

### Crystal violet staining

Following CCK-8 analysis, media was discarded and cells were fixed with 4% paraformaldehyde for 30 min, followed by washing with PBS (3 × 5 min). Crystal violet (50 μL 0.5%) was then added to each well, incubated for 15 min, followed by washing with PBS (3 × 5 min). Plates were dried at 37 °C and data recorded by microscopy photos.

### Flow cytometry

HSC-3 or CAL-33 cells were dispersed into 12-well plates and allowed to adhere overnight, followed by treatment with 0, 0.5 μM, or 1 μM LCL161 combined with/ without 10 ng/mL TNF for 24 h. Cell supernatants were collected and combined with single cell suspensions of trypsinsed cells (EDTA-free trypsin) and stained using Annexin-V FITC and PI apoptosis detection kit (Vazyme, Jiangsu, China). The data were acquired by FACs Calibur (BD Biosciences, New Jersey, USA) and analyzed using Treestar FlowJo software.

### Western blot

Treated OSCC cells were lysed at 4 °C for 30 min in RIPA buffer (Beyotime, China), containing phosphatase (Phosstop^TM^, Roche, Basel, Switzerland) and protease inhibitors (Complete^TM^, Roche, Basel, Switzerland). Samples were then centrifuged and the supernatant mixed with SDS-PAGE loading buffer, heated at 95 °C for 10 min and the extracted proteins were analysed by western blot using 4–20% SDS-PAGE (Smart Life-science, Jiangsu, China), followed by transferring to PVDF membrane (Millipore, Massachusetts, USA). Blots were blocked with 3% BSA dissolved in 1x TBST. Primary antibodies were incubated overnight on a shaker at 4 °C, followed by incubation with HRP-conjugated secondary antibodies for 45 min at room temperature and visualized by Enhanced Chemiluminescence (Vazyme, Jiangsu, China). Cytoplasmic and nuclear extracts from OSCC cell lines were prepared using NE-PER extraction reagents (Thermo Fisher Scientific Inc, Massachusetts, USA) according to the manufacturer’s instructions. Original data of WB are reported as Original Data 1.

### Immunoprecipitation (IP)

Protein A/G beads were washed 3 times with PBS containing 0.5% Triton, separated magnetically with a magnetic rack, and resuspended in 400 µL of PBS containing 0.5% Triton. Washed A/G beads were mixed with antibodies for IP for 1 h at room temperature, washed with PBS containing 0.5% Triton for 3 times, and then incubated with cell lysates for an additional 1 h at room temperature, followed by separation using DynaMag™-2 magnets (Thermo Fisher Scientific Inc, Massachusetts, USA). Beads were then washed 5 times with PBS containing 0.5% Triton and eluted with 20 μL of 1x SDS sample buffer.

### Histological preparation and IHC

Tumor tissues were fixed in 4% paraformaldehyde for a minimum of 2 days before processing for paraffin embedding. Tissue blocks were sectioned (5 μm), deparaffinized, rehydrated, and subjected to heat-induced epitope retrieval with citrate buffer, pH 6.0 (Zhongshan Golden Bridge, China), and blocked with goat serum (Zhongshan Golden Bridge, Beijing, China). Sections were stained with primary antibodies at 4 °C overnight, washed with 1x PBST for 5 min (×3), followed by incubation with anti-Mouse/Rabbit generic secondary antibodies (Maxin, Fujian, China) for 30 min at room temperature. Slides were then washed with 1x PBST for 5 min (×3) followed by incubation with DAB kit (Zhongshan Golden Bridge, Beijing, Chian) according to the manufacturer’s instructions. Then sections were counterstained, differentiated, and reblued in hematoxylin, 10% ethanol hydrochloride, and alkaline reblue solution, respectively. At last, sections were dehydrated and sealed.

Protein expression was evaluated according to a stain intensity score (0 = negative; 1 = weak; 2 = moderate; 3 = high) and a positive percentage score (0 = no positive staining, 1 = 1–25%, 2 = 26–50%, 3 = 51–75%, 4 = 76–100% positive cells). Two independent experienced pathologists performed the IHC scoring. Defined by the median final score, the expression levels of proteins were defined as “low” or “high”. Pathologists were blind to follow-up information about patients.

### H&E

Similar to the protocol of IHC, the liver and spleen tissues of mice were fixed to prepare paraffin blocks. Tissue blocks were sectioned (5 μm), deparaffinized, and rehydrated. Then sections were counterstained, differentiated, and reblued as IHC experiment, followed by staining with eosin solution. Finally, sections were dehydrated and then sealed.

### Quantitative real-time PCR

RNA from OSCC cell lines: HSC-3, HN6, CAL-33, CAL-27, and SCC-9 treated with/without 1 μM LCL161 for 24 h was extracted using the SteadyPure RNA Extraction Kit (Accurate Biology, Hunan, China) according to the manufacturer’s instructions. Detection of mRNA levels of ABC transporters followed the protocol for DNA copy number, including PCR conditions of triplicate samples. GAPDH was chosen as the reference RNA. The qPCR data was analyzed using the 2-delta delta CT method. Primer sequences were listed in Supplementary Table 6.

### ELISA

2 × 10^5^ OSCC cells were seeded into 60 mm dishes and cultured with the indicated treaments for 24 h. Supernatants were then centrifuged at 1200 r/min for 5 min to collect supernatants. A human TNF-alpha ELISA Kit (Proteintech, Wuhan, China) was used to detect TNF concentration according to the manufacturer’s instructions. Absorbance at 450 nm was measured using microplate reader (Molecular Devices, California, USA) and a four-parameter logistic curve fit was used to calculate TNF concentration.

### GC-MS analysis

HSC-3 and CAL-33 were treated with LCL161 for 6 h, followed by snap freezing with liquid nitrogen. Once liquid nitrogen had evaporated, 500 μL ddH_2_O was added to the culture plate. Samples were stored at −80 °C until GC-MS was performed. Data analysis was performed by Shengzi Biotech using methodology previously described [72].

### siRNA knockdown of ABCA3, ABCB1 and ABCB4

To knockdown ABCA3, ABCB1, and ABCB4 in SM-resistant cell lines, siRNA-ABCA3, siRNA-ABCB1, and siRNA-ABCB4 were designed and transfected into CAL-33, CAL-27, and SCC-9 with the lipofectamine 3000 transfection kit (ThermoFisher, Massachusetts, USA). The siRNA sequences had been listed in Supplementary Table 7.

### Statistical analysis

Data were presented as mean ± SD. *P* values were calculated by Student’s *t*-test, paired *t-*test, ANOVA test, chi-square test, or log-rank (Mantel–Cox) test, the *P* values were two-tailed test if no otherwise specified. Normal distribution test and homogeneity of variance test were performed if needed. Statistical analysis methods were listed in the figure captions. All results have been validated by three or more independent experiments.

## Supplementary information

Supplementary Figures and Tables

Original Data 1

## Data Availability

The data supporting the findings of this study are available from the corresponding author upon reasonable request.
