# Supplementary material for: Complex IIa formation and ABC transporters determine sensitivity of OSCC to Smac mimetics
Source: Cell Death Dis. 2024 Nov 22;15(11):855. doi: 10.1038/s41419-024-07253-w (PMC11584628; doi:10.1038/s41419-024-07253-w)
Supplement: Supplementary file 1 — Supplementary Figures and Tables [file 41419_2024_7253_MOESM1_ESM.pdf]

Supplementary Figures and Tables

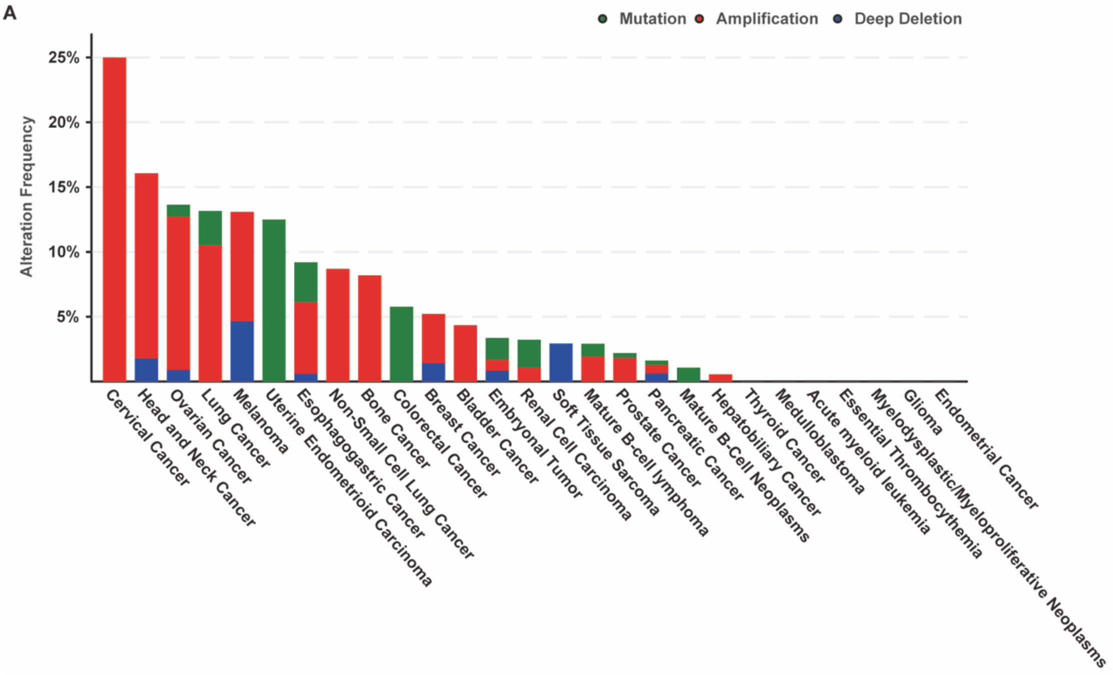

Supplementary Figure 1. Percentage of *BIRC2* and *BIRC3* genetic modifications in 27 cancers. (A) Percentage of *BIRC2* and *BIRC3* genetic modifications in 27 cancers. Data from ICGC, TCGA, Pan-cancer analysis of whole genomes, Nature 2020. Data visualization was created with the website http://www.cbioportal.org.

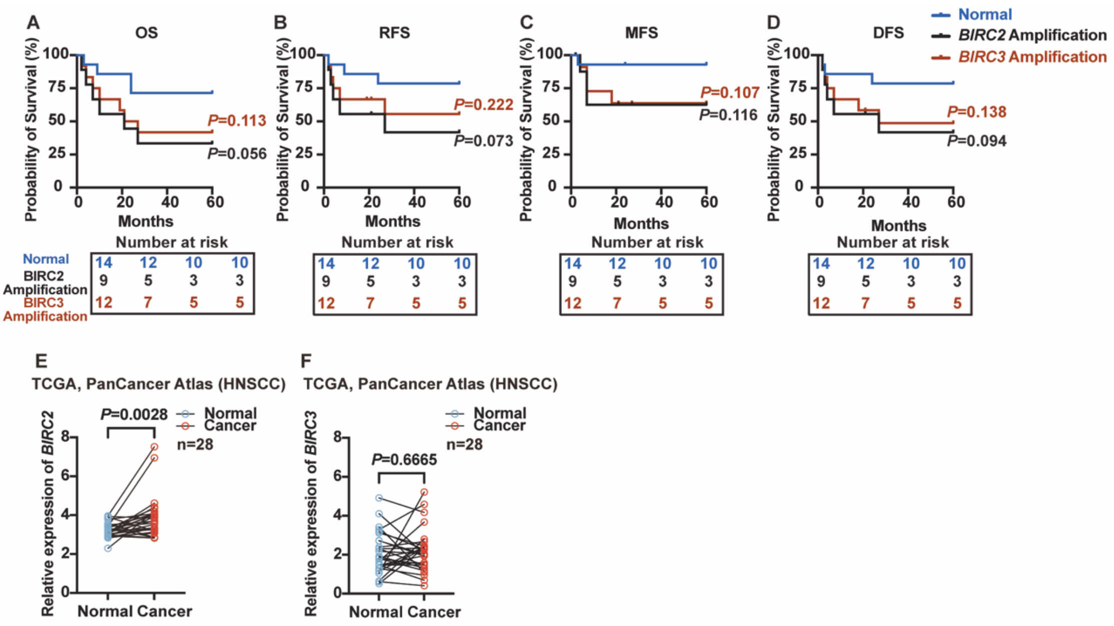

Supplementary Figure 2. The expression of *BIRC2/BIRC3* and their prognostic value. (A-D) Graphical depiction of OS (A), RFS (B), MFS (C) and DFS (D) from OSCC patients without *BIRC2/BIRC3* amplification mutations (n=14), with *BIRC2* amplification mutation (n=9), and with *BIRC3* amplification mutation (n=12), *P*=Log-rank (Mantel–Cox) test. Patient tissue sample from OSCC Clinical Sample Bank of Nanjing Stomatological Hospital. **(E, F)** Comparison of the mRNA expression of *BIRC2* **(E)** and *BIRC3* **(F)** in HNSCC tissues and normal tissues from the same patient, n=28, *P*=paired t-test. Data from TCGA, PanCancer Atlas (34).

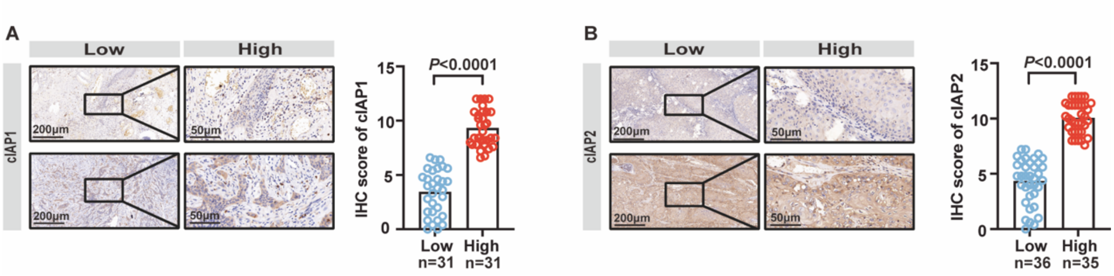

Supplementary Figure 3. Representative IHC staining of cIAP1 and cIAP2. (A, B) Representative images of low and high expression of cIAP1 (n=62) **(A)** or cIAP2 (n=71) **(B)** in OSCC tissues with graphical quantitation. *P* = t test.

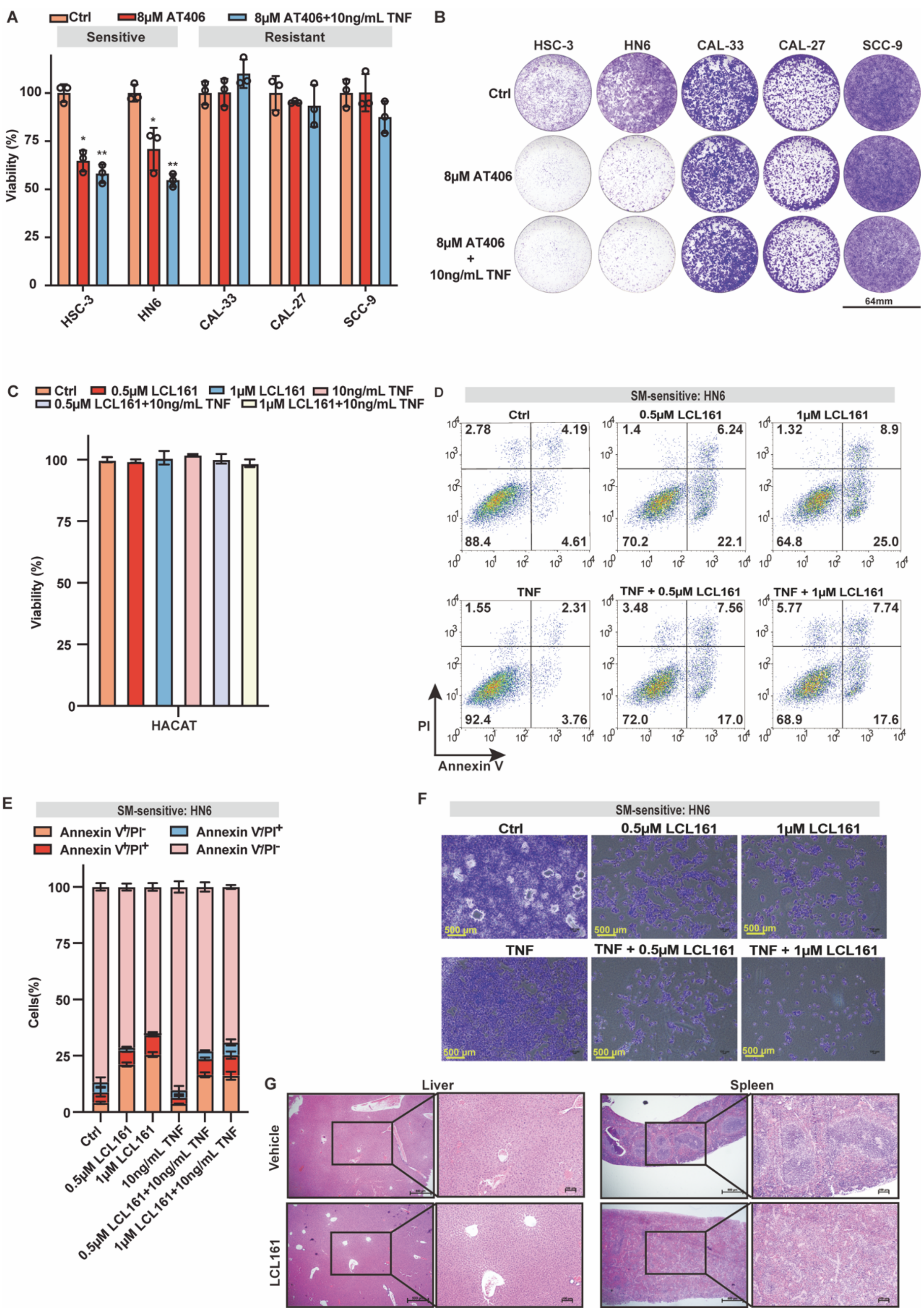

Supplementary Figure 4. The cytotoxic effects and biosafety of SMs. (A) Graphical representation of viability percentages from five OSCC cell lines after treatment with 8 μM AT406 with or without 10 ng/mL TNF, *:*P*<0.05, **:*P*<0/01, ***:*P*<0.001, *P*= ANOVA analysis. **(B)** Representative images of crystal violet staining of five OSCC cell lines after treatment with 8 μM AT406

with or without 10 ng/mL TNF for 24 hours. **(C)** Graphical representation of viability percentages from HACAT after treatment with SM (LCL161) with or without 10 ng/mL TNF for 24 hours, *:$P<0.05$, **:P<0/01, ***:$P<0.001$, P= ANOVA analysis. **(D, E)** Representative dot plots **(D)** and graphical quantitation **(E)** of the proportion of live (AV^-/PI^-), early apoptotic (AV^+/PI^-) and late apoptotic (AV^+/PI^+) cells in HN6 cells after LCL161 treatment combined with/without 10 ng/mL TNF for 24 hours. **(F)** Representative images of crystal violet staining of HN6 cells after treatment with 0.5 or 1μM LCL161 combined with or without 10 ng/mL TNF for 24 hours. **(G)** Representative images of HE staining of livers and spleens from subcutaneous xenograft model. Mice were intraperitoneal injected with Vehicle or LCL161 (20 mg/kg). Error bars are Mean ± SD.

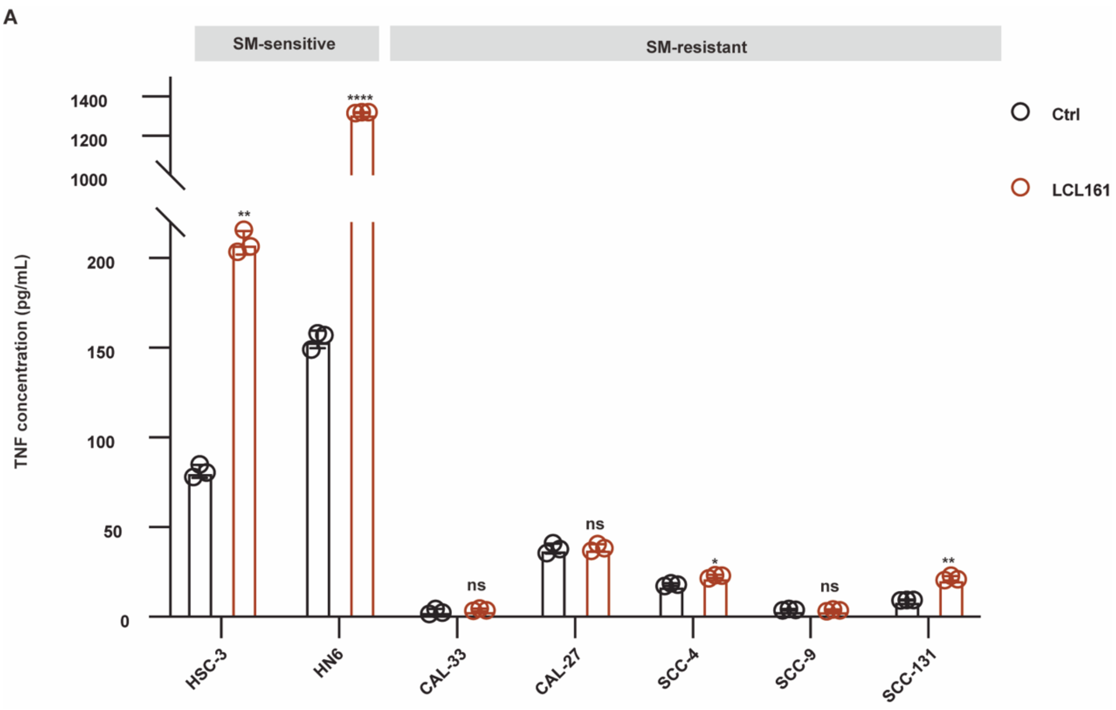

Supplementary Figure 5. SM induces sensitive cells to autocrine TNF. (A) Via ELISA to detect the concentration of TNF in cellular supernatant pre and post 24 hours treatment with 1 μM LCL161. * *P*<0.05, ** *P*<0.01, *** *P*<0.001, *** *P*<0.0001, *P*= t test.

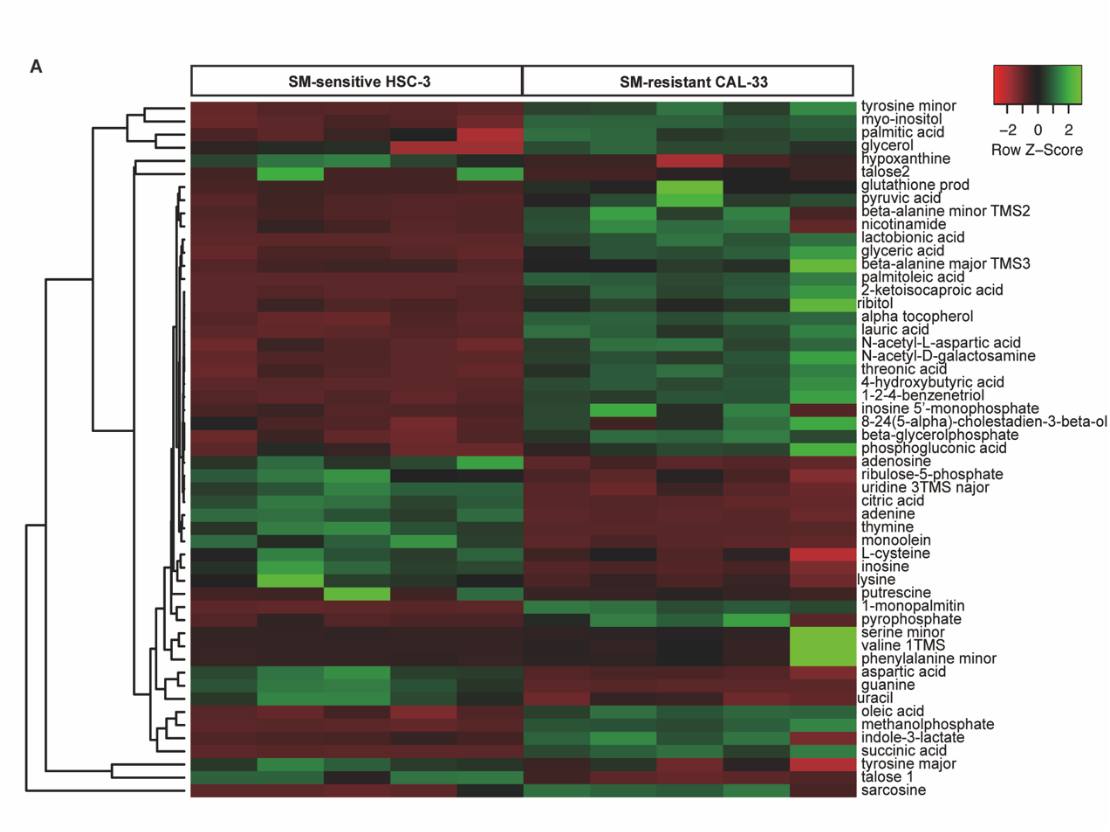

Supplementary Figure 6. The comparison of the basal metabolites between HSC-3 and CAL-33. (A) Heatmaps showing differential basal metabolite expression (variable importance for projection >1, *P*<0.05) in HSC-3 and CAL-33 OSCC cell lines.

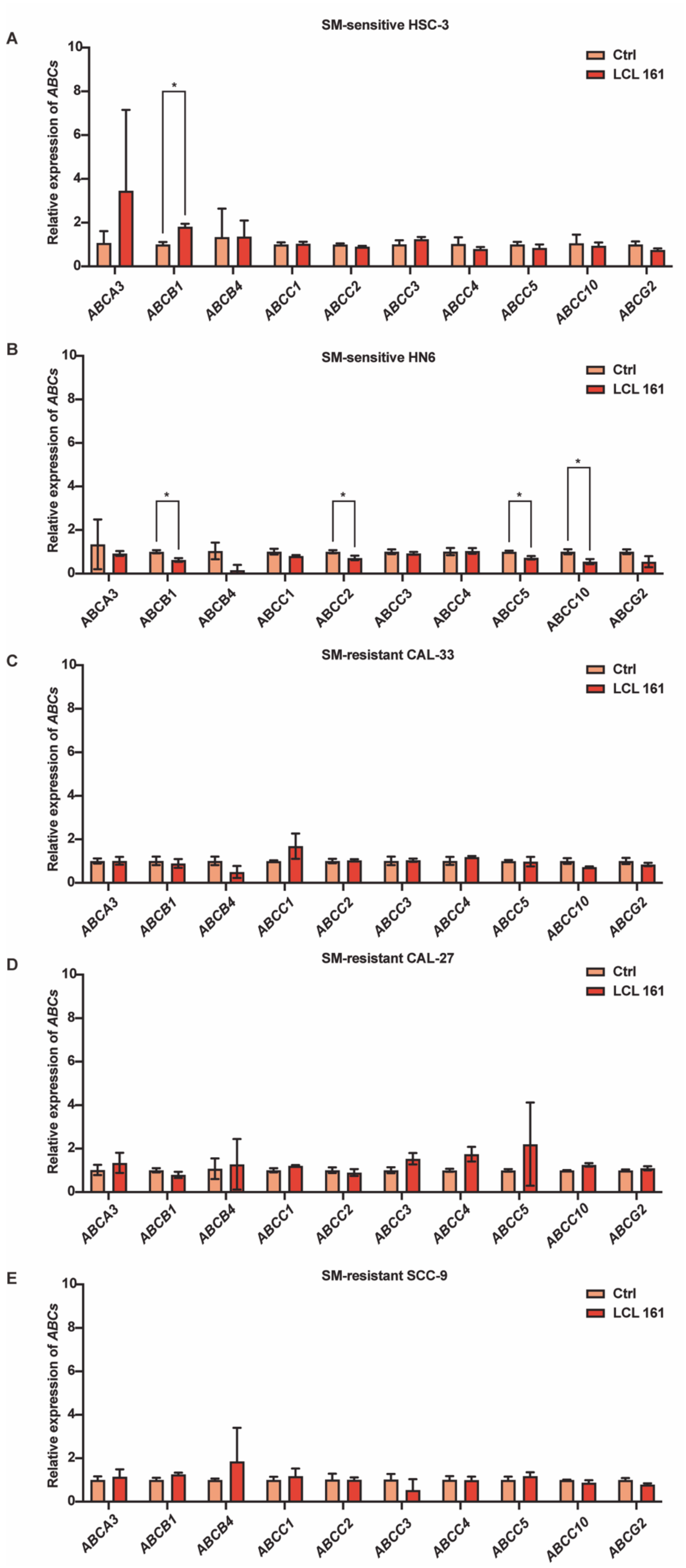

A
SM-sensitive HSC-3
B
SM-sensitive HN6
C
SM-resistant CAL-33
D
SM-resistant CAL-27
E
SM-resistant SCC-9
Ctrl
LCL 161
Relative expression of ABCs
ABCA3
ABCB1
ABCB4
ABCC1
ABCC2
ABCC3
ABCC4
ABCC5
ABCC10
ABCG2

Supplementary Figure 7. mRNA levels of ABC transporters pre and post LCL161 stimulation. (A-E) Graphical representation of the mRNA levels of ABC transporter family members in SM-sensitive and SM-resistant cells OSCC cell lines after treatment with LCL161 for 24 hours. * $P<0.05$, ** $P<0.01$, *** $P<0.001$. P= t test. Error bars are Mean ± SD.

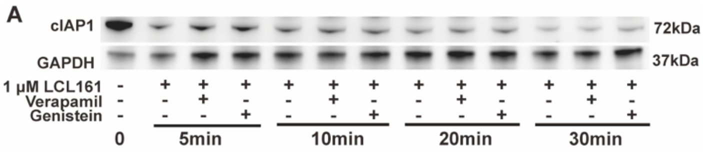

Supplementary Figure 8. The expression of cIAP1 in HN6 after LCL161 combined with ABC inhibitor treatment. (A) WB blot of the protein levels of cIAP1 in SM-sensitive HN6 after treated with LCL161 combined with genestein (5 μM) or verapamil (5 μM) for 0-30min.

Supplementary Table 1. Correlation between cIAP1 expression and clinicopathologic characteristics in OSCC patients

		cIAP1 expression in OSCC			
**Characteristics**	**Total, n**	**Low cases, n (%)**	**High cases, n (%)**	χ^2	***P***
**Gender**					
Male	36	18 (50%)	18 (50%)	0	1
Female	26	13 (50%)	13 (50%)		
**Age (years)**					
>60	38	19 (50%)	19 (50%)	0	1
≤60	24	12 (50%)	12 (50%)		
**Status**					
Dead	26	8 (30.8%)	18 (69.2%)	6.624	0.010**
Alive	36	23 (63.9%)	13 (36.1%)		
**Smoking**					
Yes	25	14 (56.0%)	11 (44.0%)	0.603	0.437
No	37	17 (45.9%)	20 (54.1%)		
**T stage**					
III-IV	26	14 (53.8%)	12 (46.2%)	0.265	0.607
I-II	36	17 (47.2%)	19 (52.8%)		
**Lymphatic metastasis**					
Yes	31	16 (51.6%)	15 (48.4%)	0.065	0.799
No	31	15 (48.4%)	16 (51.6%)		
**Recurrence**					
Yes	14	5 (35.7%)	9 (64.3%)	1.476	0.224
No	48	26 (54.2%)	22 (45.8%)		
**Metastasis**					

Yes	15	6 (40.0%)	9 (60.0%)	0.791	0.374
No	47	25 (53.2%)	22 (46.8%)		
**Differentiation**					
Moderate/ Poor	47	20 (42.6%)	27 (57.4%)	4.309	0.038*
Well	15	11 (73.3%)	4 (26.7%)		
**DOI**					
>5mm	39	16 (41.0%)	23 (59.0%)	3.387	0.066
≤5mm	23	15 (65.2%)	8 (34.8%)		
**WPOI**					
IV-V	42	15 (35.7%)	27 (64.3%)	10.629	0.001***
I-III	20	16 (80.0%)	4 (20.0%)		

*: $P<0.05$, **: $P<0.01$, ***: $P<0.001$. P= chi-square test.

Supplementary **Table 2.** Correlation between cIAP2 expression and clinicopathologic characteristics in OSCC patients

		cIAP2 expression in OSCC			
**Characteristics**	**Total, n**	**Low cases, n (%)**	**High cases, n (%)**	χ^2	***P***
**Gender**					
Male	39	22 (56.4%)	17 (43.6%)	1.127	0.288
Female	32	14 (43.8%)	18 (56.2%)		
**Age (years)**					
>60	47	24 (51.1%)	23 (48.9%)	0.007	0.932
≤60	24	12 (50%)	12 (50%)		
**Status**					
Dead	34	20 (58.8%)	14 (41.2%)	1.721	0.19
Alive	37	16 (43.2%)	21 (56.8%)		
**Smoking**					
Yes	22	11 (50.0%)	11 (50.0%)	0.006	0.937
No	49	25 (51.0%)	24 (49.0%)		
**T stage**					
III-IV	32	14 (43.8%)	18 (56.3%)	1.127	0.288
I-II	39	22 (56.4%)	17 (43.6%)		
**Lymphatic metastasis**					
Yes	34	16 (47.1%)	18 (52.9%)	0.347	0.556
No	37	20 (54.1%)	17 (45.9%)		
**Recurrence**					
Yes	17	6 (35.3%)	11 (64.7%)	2.124	0.145
No	54	30 (55.6%)	24 (44.4%)		
**Metastasis**					

Yes	15	9 (60.0%)	6 (40.0%)	0.657	0.417
No	56	27 (48.2%)	29 (51.8%)		
**Differentiation**					
Moderate/ Poor	56	25 (44.6%)	31 (55.4%)	3.389	0.048*
Well	15	11 (73.3%)	4 (26.7%)		
**DOI**					
>5mm	44	21 (47.7%)	23 (52.3%)	0.410	0.522
≤5mm	27	15 (55.6%)	12 (44.4%)		
**WPOI**					
IV-V	44	22 (50.0%)	22 (50.0%)	0.023	0.880
I-III	27	14 (51.9%)	13 (48.1%)		

*: *P*<0.05, **: *P*<0.01, ***: *P*<0.001. *P*=chi-square test.

Supplementary Table 3. Cox proportional hazards model of cIAP1 for clinicopathologic parameters in OSCC patients

	Univariate			Multivariate		
**Parameters**	**HR**	**95% CI**	***P***	**HR (95% CI)**	**95% CI**	***P***
**Gender**	1.375	0.623-3.033	0.43			
**Age (years)**	1.114	0.505-2.458	0.79			
**Smoking**	0.826	0.367-1.856	0.643			
**T stage**	1.421	0.658-3.067	0.371			
**Lymphatic**	2.882	1.276-6.508	0.011*	3.31	1.351-	0.009**
**Recurrence**	1.766	0.766-4.073	0.182			
**Distant**	3.855	1.768-8.407	0.001***	2.802	1.243-	0.013*
**Differentiation**	2.421	0.832-7.046	0.105			
**DOI**	2.867	1.147-7.167	0.024*	2.488	0.958-	0.061
**WPOI**	3.567	1.221-10.421	0.020*	2.231	0.736-	0.156
**cIAP1 expression**	2.957	1.281-6.827	0.011*	2.608	1.068-	0.035*

*: $P<0.05$, **: $P<0.01$, ***: $P<0.001$; HR: hazard ratio; CI: confidence interval.

Supplementary Table 4. Cox proportional hazards model of cIAP2 for clinicopathologic parameters in OSCC patients

	Univariate			Multivariate		
**Parameters**	**HR**	**95% CI**	***P***	**HR**	**95% CI**	***P***
**Gender**	1.819	0.899-3.678	0.096			
**Age (years)**	0.95	0.470-1.919	0.885			
**Smoking**	1.344	0.663-2.724	0.412			
**T stage**	1.635	0.830-3.219	0.155			
**Lymphatic**	4.151	1.977-8.715	0.000***	3.255	1.438-7.365	0.005**
**Recurrence**	2.245	1.122-4.490	0.022*	2.564	1.262-5.209	0.009**
**Distant metastasis**	5.239	2.571-10.675	0.000***	3.069	1.43-6.587	0.004**
**Differentiation**	1.898	0.734-4.907	0.186			
**DOI**	1.566	0.746-3.286	0.236			
**WPOI**	1.453	0.705-2.995	0.312			
**cIAP2 expression**	0.614	0.310-1.217	0.162			

*: *P*<0.05, **: *P*<0.01, ***: *P*<0.001.

Supplementary Table 5. Details of commercially sourced antibodies

Antibody	Company	Product No.	Species
cIAP1	Abcam, Cambridgeshire, England	108361	Rabbit
cIAP2	Abcam, Cambridgeshire, England	32059	Rabbit
caspase-3	CST, Massachusetts, USA	9662	Rabbit
cleaved-caspase-3	CST, Massachusetts, USA	9664	Rabbit
caspase-8	CST, Massachusetts, USA	9746	Mouse
p-p65	CST, Massachusetts, USA	3033	Rabbit
RIP1	CST, Massachusetts, USA	3493	Rabbit
FADD	Abcam, Cambridgeshire, England	108601	Rabbit
p100/p52	CST, Massachusetts, USA	37359	Rabbit
p105/p50	CST, Massachusetts, USA	3035	Rabbit
GAPDH	Immunoway, Californian, USA	YM3029	Mouse
LAMIN B1	CST, Massachusetts, USA	13435	Rabbit
α Tublin	CST, Massachusetts, USA	2125	Rabbit
β Actin	CST, Massachusetts, USA	4967	Rabbit
NIK	Abclonal, Wuhan, China	A11585	Rabbit
p-p65	CST, Massachusetts, USA	8242	Rabbit
ABCA3	Abclonal, Wuhan, China	A6862	Rabbit
ABCB1	Proteintech, Wuhan, China	22336	Rabbit
ABCB4	Proteintech, Wuhan, China	27726	Rabbit

Supplementary Table 6. Primer sequences.

Primer name	Primer sequence (5' to 3')
BIRC2-F	AGCACGATCTTGTCAGATTGG
BIRC2-R	GGCGGGGAAAGTTGAATATGTA
BIRC3-F	AAGCTACCTCTCAGCCTACTTT
BIRC3-R	CCACTGTTTTCTGTACCCGGA
Globin-F	TCTTCACGGTGTACCCCAG
Globin-R	ATTAGCAGCGGAAAGTTGGCT
GAPDH-F	TGGTGAAGACGCCAGTGGA
GAPDH-R	GCACCGTCAAGGCTGAGAAC
ABCA3-F	CTCCTCTGGAAGAACTACACCC
ABCA3-R	GGGCACATTTTCCGACTGAATC
ABCB1-F	GGGATGGTCAGTGTTGATGGA
ABCB1-R	GCTATCGTGGTGGCAAACAATA
ABCB4-F	ATAGCTCACGGATCAGGTCTC
ABCB4-R	GGATTTAGCAGCGACAAGGAAA
ABCC1-F	TTACTCATTCAGCTCGTCTTGTC
ABCC1-R	CAGGGATTAGGGTCGTGGAT
ABCC2-F	TCTCTCGATACTCTGTGGCAC
ABCC2-R	CTGGAATCCGTAGGAGATGAAGA
ABCC3-F	CACCAACTCAGTCAAACGTGC
ABCC3-R	GCAAGACCATGAAAGCGACTC

ABCC4-F	AGCTGAGAATGACGCACAGAA
ABCC4-R	ATATGGGCTGGATTACTTTGGC
ABCC5-F	ATCATGGCTTGAGTGCTCTGA
ABCC5-R	AGACCACACGTCTTCCATTGA
ABCC10-F	GTCCAGATTACATCCTACCCTGC
ABCC10-R	GCCAACACCTCTAGCCCTATG
ABCG2-F	ACGAACGGATTAACAGGGTCA
ABCG2-R	CTCCAGACACACCACGGAT

Supplementary Table 7. siRNA sequences.

Oligo name	5' to 3'
ABCA3-human-2240-S	CGCUGUUCCUCAAGCAGAATT
ABCA3-human-2240-A	UUCUGCUUGAGGAACAGCGTT
ABCA3-human-457-S	CACCUACGGUUCAGUUACATT
ABCA3-human-457-A	UGUAACUGAACCGUAGGUGTT
ABCA3-human-4026-S	GCGGACACUGACAGAAUUATT
ABCA3-human-4026-A	UAAUUCUGUCAGUGUCCGCTT
ABCB1-human-2240-S	CACCCAGGCAAUGAUGAUUTT
ABCB1-human-2240-A	AUACAUCAUUGCCUGGGUGTT
ABCB1-human-2240-S	GCGAAGCAGUGGUUCAGGUTT
ABCB1-human-2240-A	ACCUGAACCACUGCUUCGCTT
ABCB1-human-2240-S	CACCCGACUUACAGAUGAUTT
ABCB1-human-2240-A	AUCAUCUGUAAGUCGGGUGTT
ABCB4-human-2240-S	GGCGAGAUCCUCACCAGAATT
ABCB4-human-2240-A	UUCUGGUGAGGAUCUCGCCTT
ABCB4-human-2240-S	GGUUGGAAUGUUCUUUCAATT
ABCB4-human-2240-A	UUGAAAGAACAUUCCAACCTT
ABCB4-human-2240-S	CCAGAAUGCAGACUUAAUATT
ABCB4-human-2240-A	UAUUAAGUCUGCAUUCUGGTT
siRNA-FAM-negative-S	UUCUCCGAACGUGUCACGUTT
siRNA-FAM-negative-A	ACGUGACACGUUCGGAGAATT

GAPDH-human-S	GUAUGACAACAGCCUCAAGTT
GAPDH-human-A	CUUGAGGCUGUUGUCAUACTT
siRNA-negative-S	UUCUCCGAACGUGUCACGUTT
siRNA-negative-A	ACGUGACACGUUCGGAGAATT
