# Supplementary material for: Complex IIa formation and ABC transporters determine sensitivity of OSCC to Smac mimetics
Source: Cell Death Dis. 2024 Nov 22;15(11):855. doi: 10.1038/s41419-024-07253-w (PMC11584628; doi:10.1038/s41419-024-07253-w)

Figure 4A

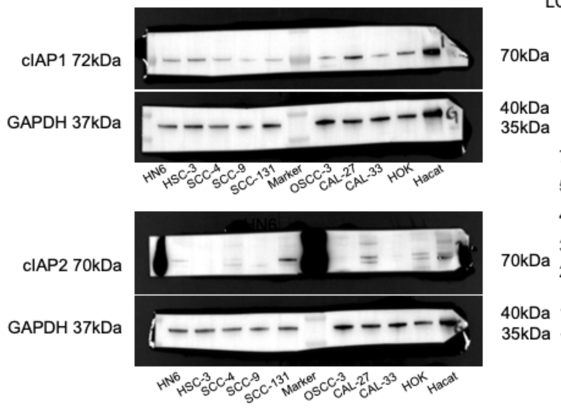

Figure 4B HSC-3

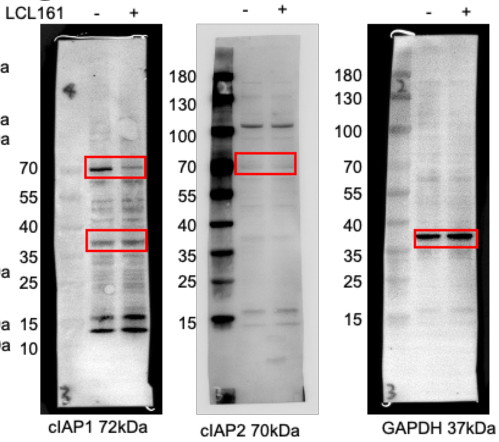

Figure 4B HN6

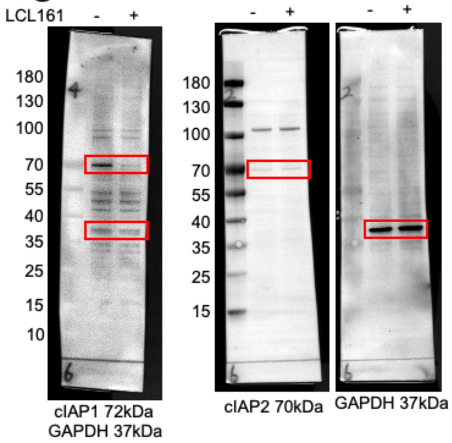

Figure 4B CAL-33

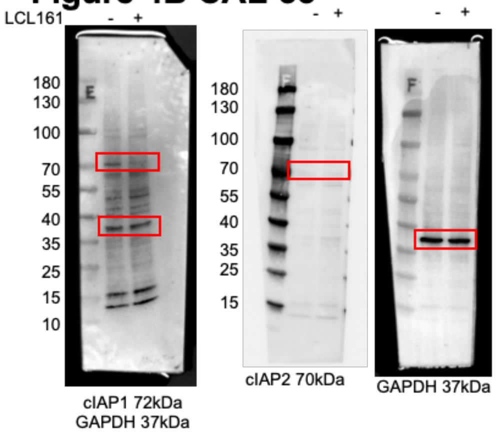

Figure 4B SCC-9

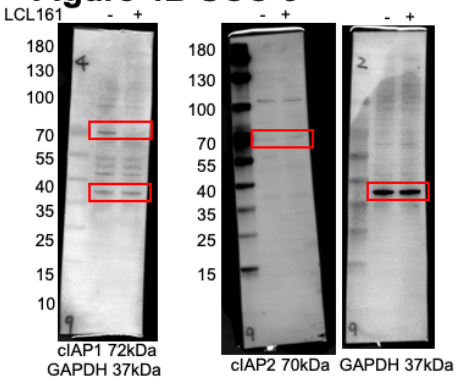

Figure 5C

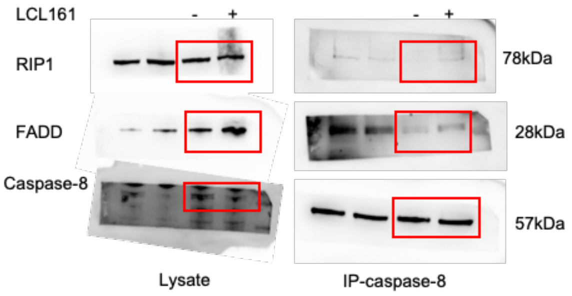

Figure 5D

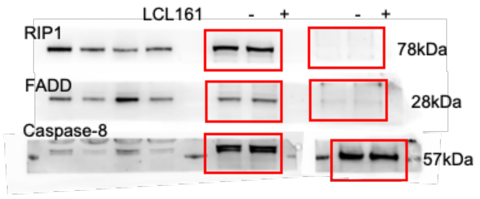

Figure 5E

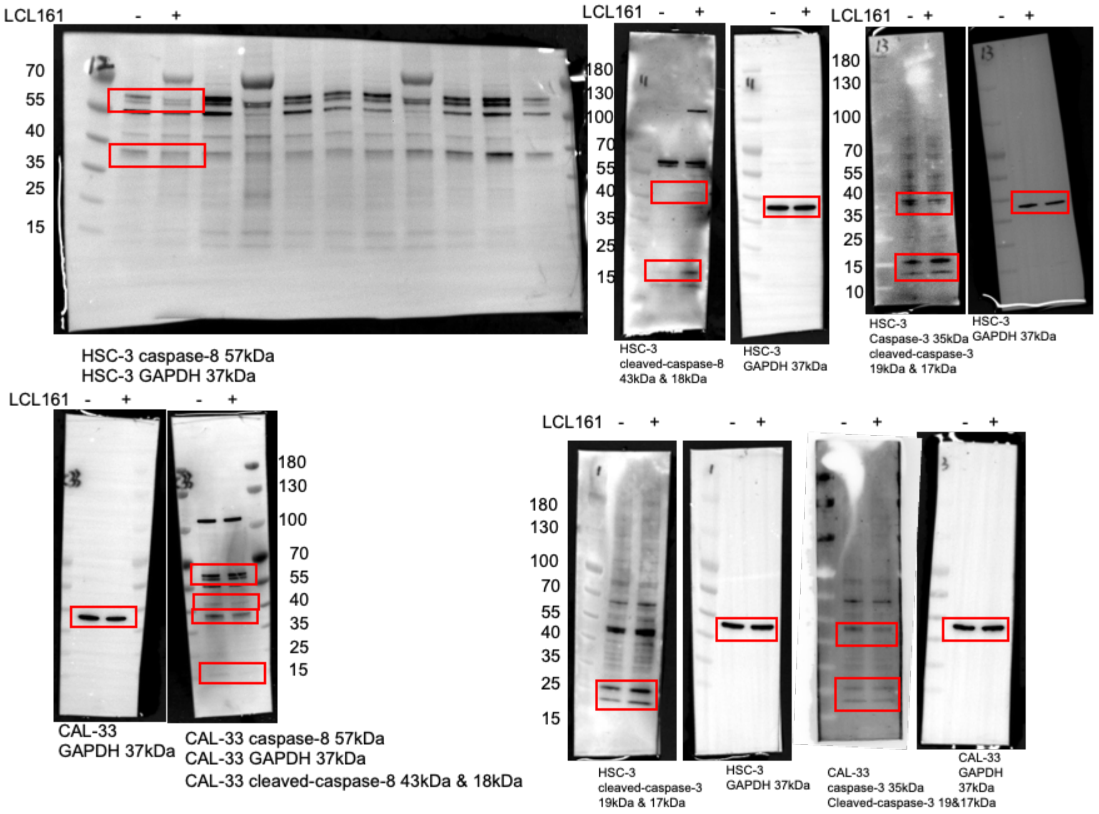

Figure 6A

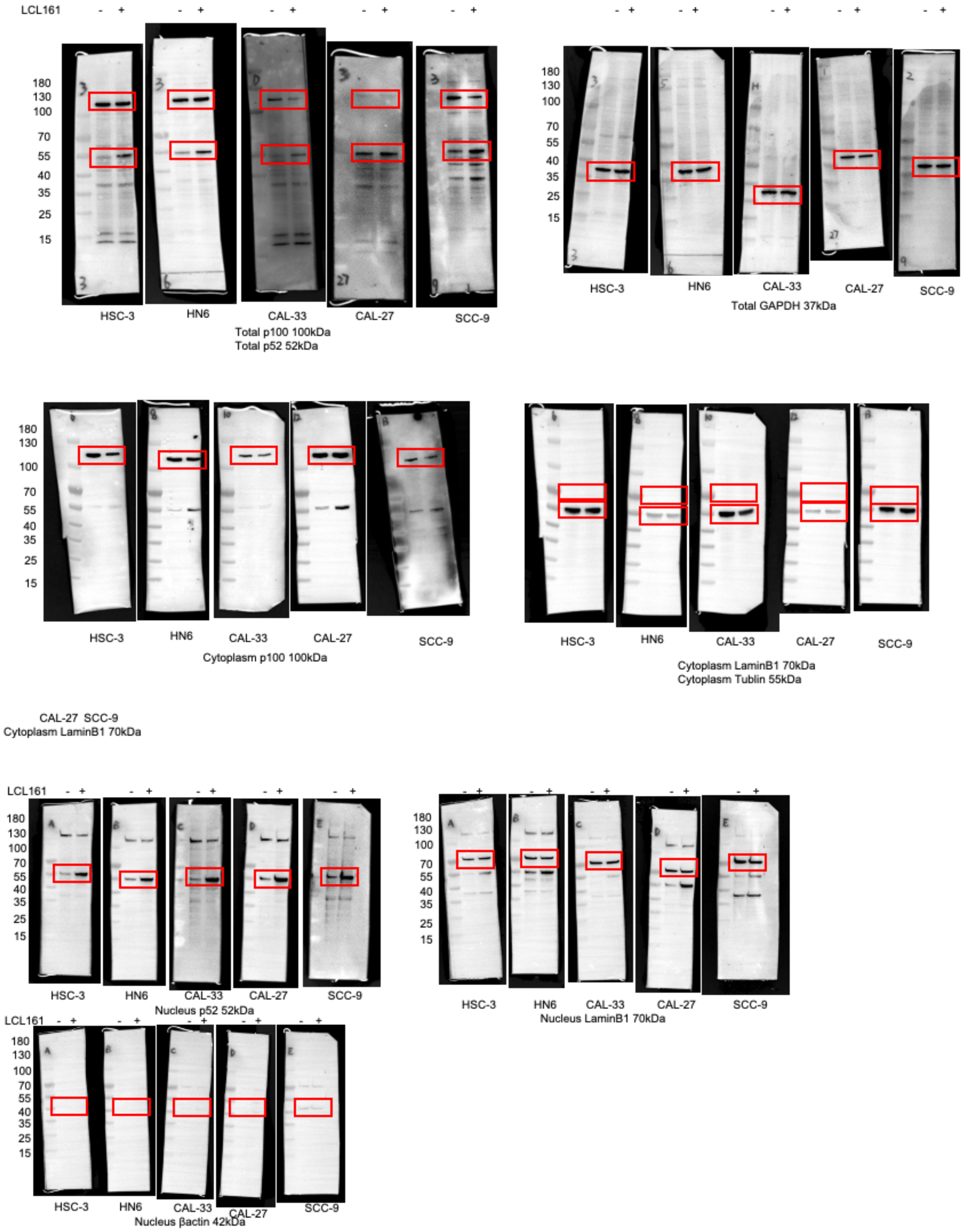

LCL161
- +
180
130
100
70
55
40
35
25
15
HSC-3
HN6
CAL-33
CAL-27
SCC-9
Total p100 100kDa
Total p52 52kDa
Total GAPDH 37kDa
Cytoplasm p100 100kDa
Cytoplasm LaminB1 70kDa
Cytoplasm Tublin 55kDa
CAL-27 SCC-9
Cytoplasm LaminB1 70kDa
Nucleus p52 52kDa
Nucleus LaminB1 70kDa
Nucleus βactin 42kDa

Figure 6D

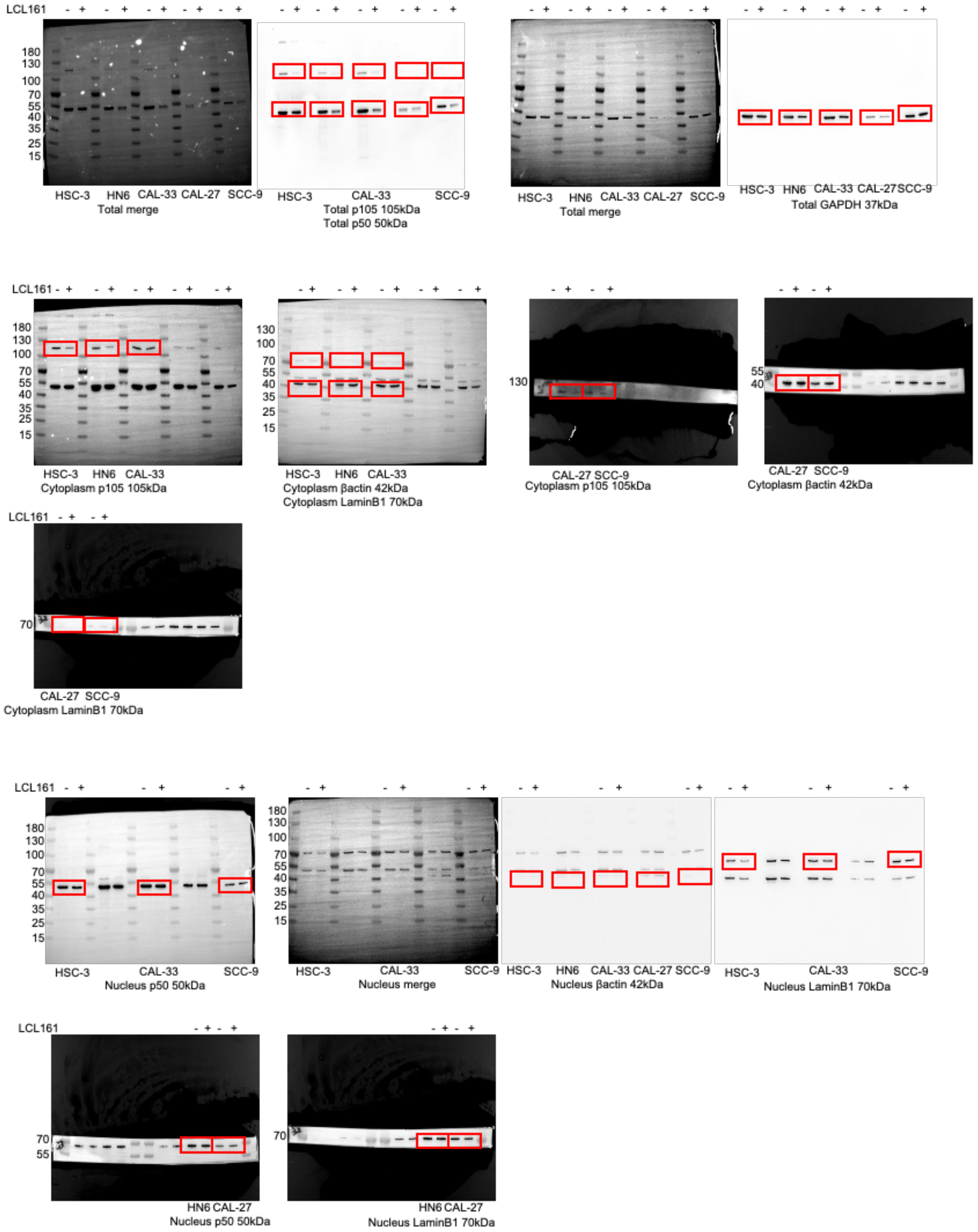
LCL161
Total merge
Total p105 105kDa
Total p50 50kDa
Total merge
Total GAPDH 37kDa
Cytoplasm p105 105kDa
Cytoplasm βactin 42kDa
Cytoplasm LaminB1 70kDa
Cytoplasm p105 105kDa
Cytoplasm βactin 42kDa
Cytoplasm LaminB1 70kDa
Nucleus p50 50kDa
Nucleus merge
Nucleus βactin 42kDa
Nucleus LaminB1 70kDa
Nucleus p50 50kDa
Nucleus LaminB1 70kDa
HSC-3
HN6
CAL-33
CAL-27
SCC-9

Figure 8I

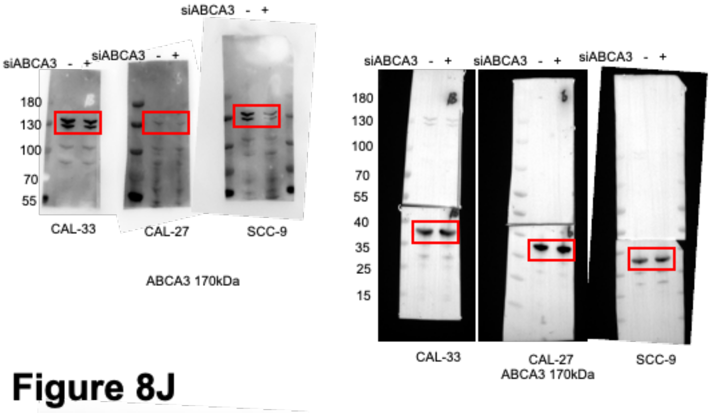

Figure 8J

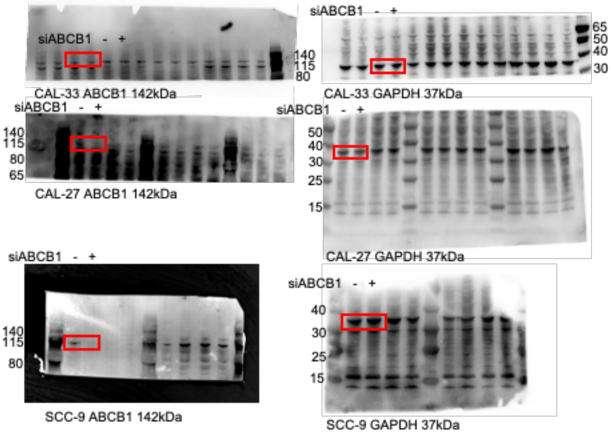

Figure 8K

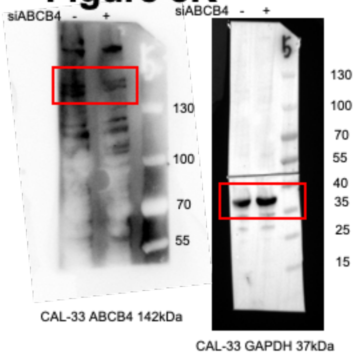

Figure 8K

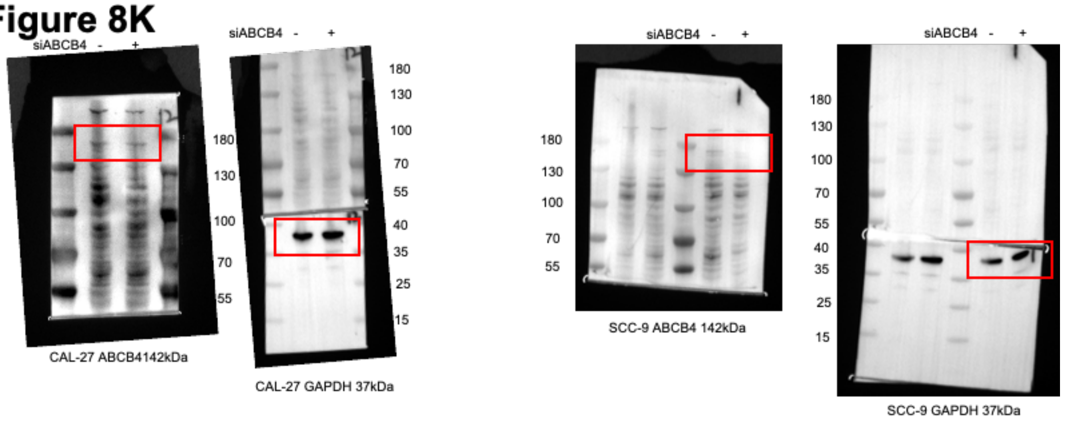

Figure 8O

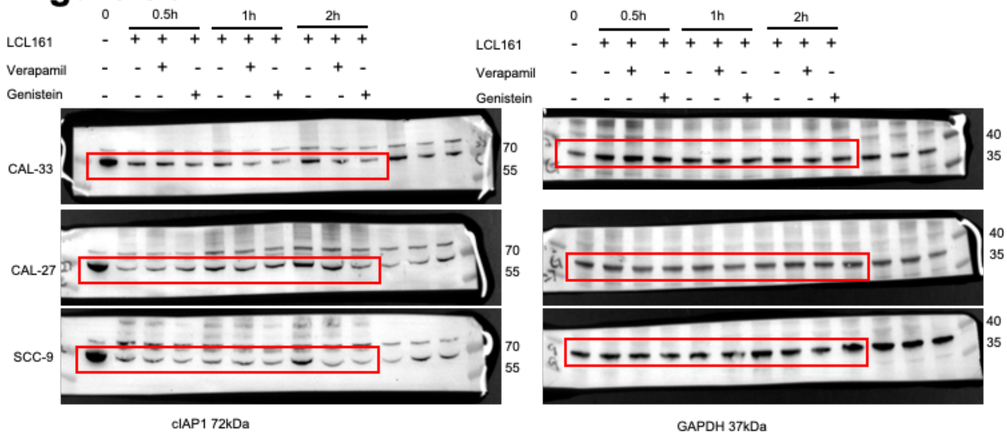

Figure 8P

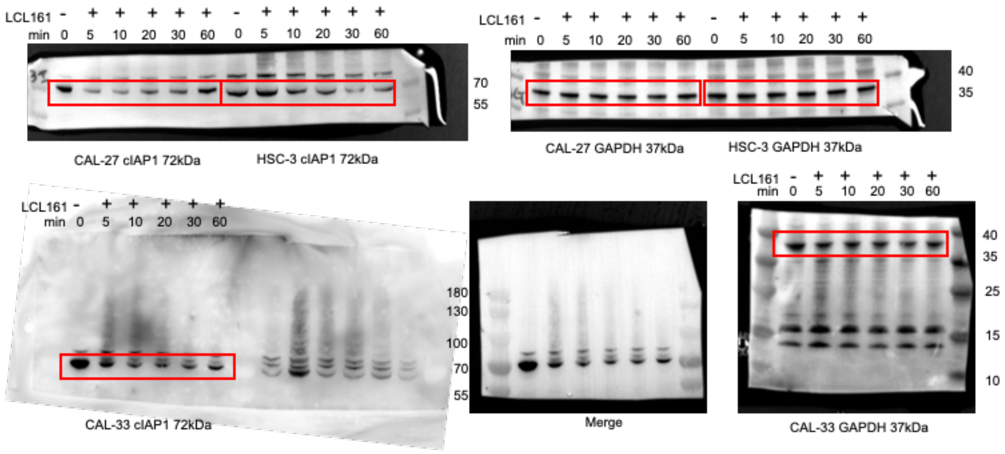

Supplementary Figure 8A

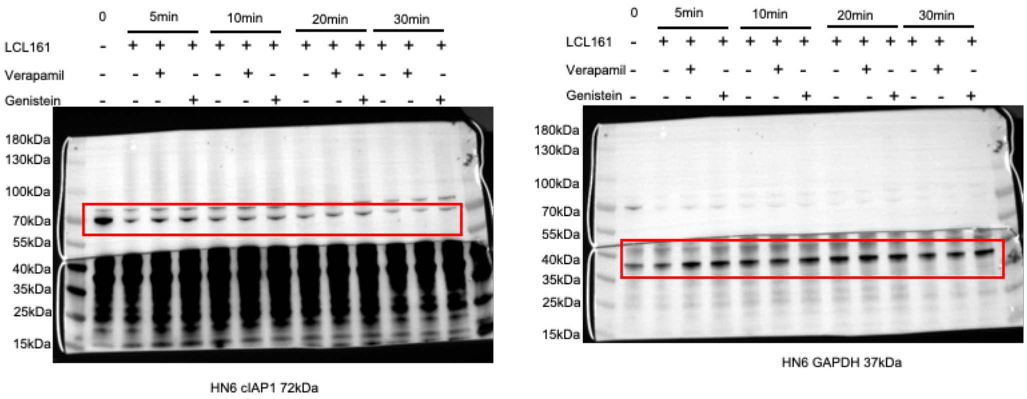

Supplement: Supplementary file 2 — Original Data 1 [file 41419_2024_7253_MOESM2_ESM.pdf]
